# Changes in tumor and cardiac metabolism upon immune checkpoint

**DOI:** 10.1007/s00395-024-01092-8

**Published:** 2024-12-10

**Authors:** Anna-Sophia Leven, Natalie Wagner, Stephan Nienaber, Daniel Messiha, Alpaslan Tasdogan, Selma Ugurel

**Affiliations:** 1 Department of Dermatology, Venereology and Allergology, University Hospital Essen, University Duisburg-Essen, Essen, Germany; 2 Clinic III for Internal Medicine, University of Cologne, Faculty of Medicine and University Hospital Cologne, Cologne, Germany; 3 Department of Cardiology and Vascular Medicine, West German Heart and Vascular Centre, University of Duisburg-Essen, Essen, Germany; 4 German Cancer Consortium (DKTK), Partner Site Essen/Düsseldorf, Essen, Germany; 5 National Center for Tumor Diseases (NCT)-West, Campus Essen, and Research Alliance Ruhr, Research Center One Health, University Duisburg-Essen, Essen, Germany

**Keywords:** Immune-checkpoint inhibition, Cardiac metabolism, Cancer metabolism, Anti-cancer therapy

## Abstract

Cardiovascular disease and cancer are the leading causes of death in the Western world. The associated risk factors are increased by smoking, hypertension, diabetes, sedentary lifestyle, aging, unbalanced diet, and alcohol consumption. Therefore, the study of cellular metabolism has become of increasing importance, with current research focusing on the alterations and adjustments of the metabolism of cancer patients. This may also affect the efficacy and tolerability of anti-cancer therapies such as immune-checkpoint inhibition (ICI). This review will focus on metabolic adaptations and their consequences for various cell types, including cancer cells, cardiac myocytes, and immune cells. Focusing on ICI, we illustrate how anti-cancer therapies interact with metabolism. In addition to the desired tumor response, we highlight that ICI can also lead to a variety of side effects that may impact metabolism or vice versa. With regard to the cardiovascular system, ICI-induced cardiotoxicity is increasingly recognized as one of the most life-threatening adverse events with a mortality of up to 50%. As such, significant efforts are being made to assess the specific interactions and associated metabolic changes associated with ICIs to improve both efficacy and management of side effects.

## Introduction

Cancer and cardiovascular diseases are the most common reasons for death in the western world. The risks of these diseases are known to be increased due to smoking, arterial hypertension, diabetes, sedentary lifestyle, aging, unbalanced diet, and alcohol consumption [92].

In this context, the study of cellular metabolism is becoming increasingly important with current research focusing on changes and adaptations of metabolism in cancer patients. This may also affect the efficacy and tolerability of anti-tumor therapies such as immune-checkpoint inhibition (ICI).

In this review, we highlight the cellular metabolism and its adaptations upon ICI therapy in cancer cells and cardiomyocytes as well as in immune cells. We shed light on the interplay among anti-tumor therapies, with a focus on ICI. In addition to the desired anti-tumor response, ICI like other cancer therapies can cause treatment-induced side effects. These are mostly immune-related, can vary widely in severity and duration, and can affect at least every organ system. With regard to the cardiovascular system, ICI-induced cardiotoxicity is increasingly being recognized for its high acuity and life-threatening outcome, with a mortality of up to 50% [78]. Therefore, there is currently a great effort to assess the specific interactions and corresponding metabolic changes in regard to ICI to improve efficacy as well as management of adverse side effects.

## Immunotherapy and cancer

In recent years, the introduction of ICI has revolutionized the treatment of many types of solid and hematological cancers. Currently, a total of seven monoclonal antibodies against PD-1 (programmed cell death protein 1) or its ligand PD-L1 (programmed cell death 1 ligand 1), and two antibodies against CTLA-4 (cytotoxic T-lymphocyte-associated protein 4) have been approved by the Food and Drug Administration (FDA) and European Medicines Agency (EMA) for anti-cancer therapy in more than 85 indications [8]. As a new and additional ICI target LAG-3 (lymphocyte-activation gene 3) was recently introduced with high expectations for an improved treatment outcome, particularly as a combination therapy of LAG-3 and PD-1 antibodies due to promising results of the phase-III trial RELATIVITY-047 [152]. Another ICI target under study is TIGIT (T-cell immunoreceptor with immunoglobulin and ITIM domain), which was described to lead to a favorable treatment outcome in combined ICI. A current phase-III trial studying pembrolizumab (PD-1) plus or minus an anti-TIGIT antibody in melanoma patients in the adjuvant setting has just completed recruitment and showed that the study was unlikely to meet its primary endpoint, with more patients discontinuing treatment in the coformulation arm than in the pembrolizumab arm (NCT05665595) [144].

**Fig. 1:**
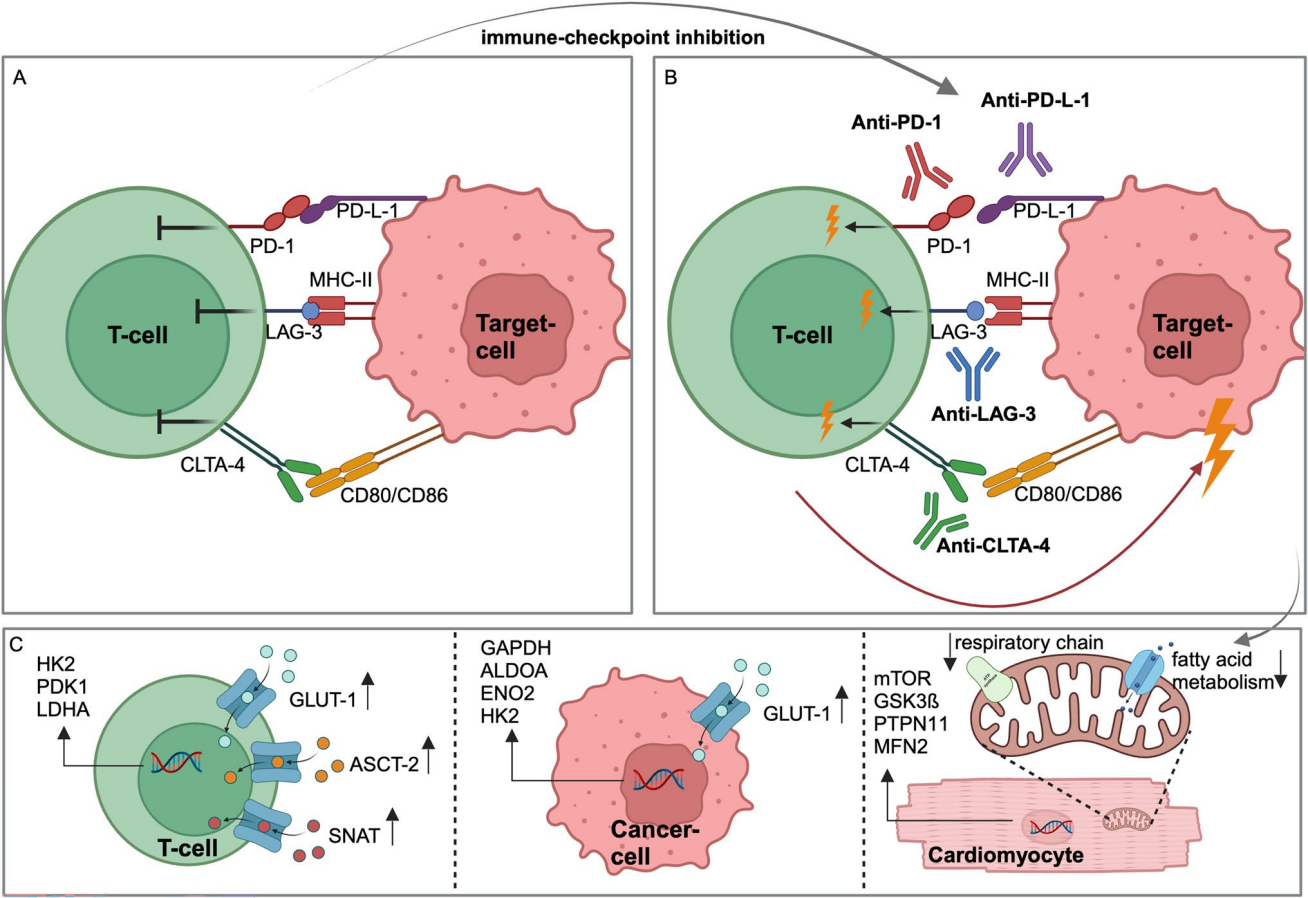
Mechanisms of immune-checkpoint inhibition. Binding of PD-1, LAG-3, and CTLA-4 inhibits T-cell activation and immune response (**A**). Immune-checkpoint inhibition by anti-PD1, anti-LAG-3, and anti-CLTA-4 allows T-cell activation (**B**). This results in changes in T-cell metabolism and gene transcription, as well as anti-tumor response and potentially cardiovascular immune-related adverse events (**C**). Figure created with Biorender.com

As T cells are the primary target of ICI therapy, the major goal is to modulate the patient's own immune system by blocking the interaction between checkpoint receptor–ligand molecules and T cells. This enhances the recognition and subsequent destruction of cancer cells by T cells, as illustrated in Fig. 1. However, the response rates are highly variable and not all cancer patients’ benefit from ICI therapy. It is therefore of both—scientific and clinical interest—to identify patient characteristics that may predict an individual's response or resistance to ICI. An important aspect to consider is the occurrence of immune-related adverse events (irAEs), which are caused by an immune reaction directed against the body’s own benign cells and tissues, including the heart. These irAEs can occur during or after ICI therapy [52]. In addition to metabolic factors, genetic factors and patient-specific factors, such as personal diet, gut microbiome, gender, and lifestyle, are increasingly being discussed as potential factors influencing response, outcome, and adverse events upon ICI therapy [178].

### Immune-related adverse events among immunotherapy

The frequency and severity of immune-related adverse events (irAEs) upon ICIs can vary significantly based on the treatment regimen, combinations of agents, and dosages used [57, 70]. The organs most commonly affected by irAEs are the skin, intestines, liver, endocrine organs, and lungs [9, 40, 57, 146, 178]. The severity of side effects can range from mild to moderate to severe and even fatal [58]. Typically, irAEs occur during the first 12 weeks of ICI treatment, but can also manifest with a time delay, and even become evident only after treatment discontinuation. Therefore, it is important to closely and regularly monitor all patients who have received ICIs for any unusual symptoms that might be ICI-related. Almost every organ system can be affected by the side effects of ICI therapy. Depending on the severity and organ manifestation, multidisciplinary collaboration is recommended to diagnose and treat these irAEs appropriately. In this review, we will focus on the most common irAEs, as well as on those affecting the cardiovascular system due to their high mortality.

The most common irAEs include diarrhea, colitis, fatigue, elevated liver enzymes up to fulminant hepatitis, and inflammation of the endocrine organs, such as thyroiditis, hypophysitis, or pancreatitis, potentially leading to diabetes mellitus [99]. Although the majority of irAEs are reversible with appropriate treatment, the endocrine and cardiac irAEs in particular may lead to permanent organ destruction or cause long-term metabolic effects. Treatment of these side effects usually involves discontinuation of ICI therapy and initiation of an immunosuppressive therapy, initially most commonly with systemic corticosteroids. If the condition is steroid-refractory, other immunosuppressive drugs such as anti-TNFα antibodies or mycophenolate mofetil may additionally be needed [70]. Recent studies suggested that a more tailored approach with Ruxolitinib and Abatacept may be more effective in cases of severe myocarditis [137, 169]. Given that metabolism is a complex interplay of many factors, it is reasonable to assume that, in addition to metabolic changes caused by ICI therapy, both the irAEs and their management could also impact on cellular metabolism.

### Cardiac immune-related adverse events

With regard to the specific interest of this review, we would like to focus on cardiac irAE in the context of ICI therapy. The underlying mechanisms of cardiotoxicity by ICI, and cardioprotective approaches during cancer therapy are discussed in more detail in other articles in this special issue.

The broad topic of cardiac irAEs caused by ICIs can be categorized according to the time of clinical presentation. Early onset cardiovascular AEs include a non-inflammatory cardiac dysfunction, pericarditis, arrhythmia, and acute coronary syndrome [50]. The manifestation of myocarditis can vary from subclinical to fulminant, with acute left ventricle dysfunction and ventricular arrhythmia [96, 138]. Similarly, non-inflammatory cardiac dysfunction can range from asymptomatic left-ventricular dysfunction to acute heart failure [92, 135].

Although late-onset cardiovascular adverse events after ICI treatment commonly are neither fulminant nor cause immediate harm to patients, current evidence suggests that they account for a large and previously underestimated proportion of ICI-induced cardiovascular adverse events [79]. Long-term cardiovascular effects of ICI therapy can include late-onset cardiac dysfunction. This may present as asymptomatic cardiac dysfunction, or as symptomatic heart failure, which can manifest as either reduced (HFrEF) or preserved ejection fraction (HFpEF). In addition, ICI therapy may lead to progression of atherosclerotic cardiovascular disease [50].

Myocarditis caused by ICI therapy is a rare, but serious complication associated with a high mortality rate of 27% and 46%. Therefore, it is the most lethal form of ICI-related cardiotoxicity [41, 106]. Affected patients most commonly develop symptoms within the first three months of ICI treatment and present with or without reduced ejection fraction [96]. ICI-associated myocarditis is associated with an infiltration of the myocardium by CD4 + and CD8 + T cells and CD68 + macrophages. Interestingly, identical T lymphocyte clones have been found in the myocardium, skeletal muscle, and tumor tissue of the same patient. This might be due to cross-reactivity of tumor antigens with antigens on other host cells [68]. The resulting T-cell infiltration initiates the activation of a cascade of downstream pro-inflammatory cytokines, such as IL-1a, IL-2, IFNα2, and IL-17, which in turn drive myocardial inflammation [6, 51, 103]. Because PD-1-deficient mice showed elevated levels of troponin I autoantibodies, it has been proposed that this mechanism might be responsible for ICI-related myocarditis. However, specific autoantibodies have not yet been identified and further evaluation of risk factors for ICI-induced myocarditis requires ongoing research [117, 138]. Recent data have shown that troponin T antibodies are associated with MACE. A normal troponin I level in the setting of clinically suspected ICI-related myocarditis should prompt a troponin T test to further validate the diagnosis. The exact correlation between circulating troponin levels and severity of ICI-related myocarditis is still not fully elucidated [10, 82].

In addition to ICI-induced myocarditis, numerous cases of ICI-induced cardiac dysfunction have been reported, including asymptomatic left-ventricular systolic dysfunction, Takotsubo cardiomyopathy, and heart failure independent of myocarditis. Early studies suggest that left-ventricular systolic dysfunction and Takotsubo cardiomyopathy account for a large proportion of ICI-related cardiac irAEs [1]. However, the reported incidence of ICI-induced cardiac dysfunction varies widely between trials and remains to be prospectively determined as echocardiography has not been routinely used in oncology trials. Therefore, it is assumed that cardiac dysfunction or heart failure following ICI treatment is more common than previously thought and may be underdiagnosed in clinical practice. Prospective studies that include careful assessment of cardiac function before, during, and after ICI treatment are essential to assess the real incidence and will increase the overall understanding of cardiac dysfunction after ICI therapy [50, 79, 92]. Laenens and colleagues reported on the incidence of major adverse cardiovascular events (MACE) in patients treated with ICIs. The majority of patients (43.8%) had HFpEF, 31.3% had an asymptomatic decline in left-ventricular systolic function, 18.8% had HfrEF, and 6.3% had Takotsubo cardiomyopathy [50, 79]. Importantly, this study was the first to demonstrate the high incidence of HFpEF following ICI therapy.

It is widely accepted that heart failure after ICI therapy may be a late-onset event, occurring months or even years after initial oncologic treatment. Protracted subclinical myocarditis after ICI therapy and residual cardiomyopathy after recovery from acute myocarditis may be potential mechanisms of late-onset heart failure [69, 115]. In addition, treatment with ICI may lead to dyslipidemia, which may result in the progression of atherosclerotic cardiovascular disease and, in the long term, ischemic heart failure [35, 37, 50, 147]. Although the development of acute coronary syndrome (ACS) is an extremely rare ICI-associated cardiovascular complication, particularly observed after treatment with atezolizumab or pembrolizumab, it has been hypothesized that ICI treatment may lead to coronary spasm or coronary vasculitis as a result of a hyperinflammatory state [21, 29, 39]. In a mouse model using PD1-deficient and low-density lipoprotein receptor knockout mice, the progression of atherosclerotic lesions was accelerated [93]. Thus, it is possible that ICI-related ACS is driven by destabilization of existing coronary atherosclerotic plaques, ultimately leading to plaque rupture and myocardial infarction [29, 184].

The exact underlying pathomechanisms of ICI-related cardiotoxicity are still under investigation, but a growing body of evidence points toward to CD4 + T-cell-mediated inflammation [6]. Interestingly, unlike cardiotoxic drugs such as anthracyclines, ICIs do not mediate direct cardiotoxicity through cardiomyocyte apoptosis, but increase pro-inflammatory CD4 + T-cell-mediated cytokine production. This is mainly driven by the production of pro-inflammatory cytokines (TNF-α, granzyme B, IFN-γ) and transcription factors (NLRP3, MyD88, and p65/NF-κB) [130, 184]. Diabetes, obesity, and ICI combination therapy have been identified as the main risk factors that increase the risk and severity of ICI-related cardiotoxicity [113, 173, 186]. Further research is warranted to further elucidate the underlying pathomechanisms of ICI-related cardiotoxicity including metabolism to enable prevention, timely diagnosis, and appropriate treatment of ICI-related cardiac complications.

### Cell and tumor metabolism

In addition to the regulation and adaptation of the immune system, metabolic mechanisms in immune and cancer cells have emerged as an important influencing factor in the context of tumor diseases and their treatment. In recent years, this topic has garnered increasing attention and continues to be the focus of ongoing research. Metabolism plays a crucial role in all cell functions by controlling the production of adenosine triphosphate (ATP). ATP is the main fuel for cellular activity and plays a central role in several diseases, including cancer. In addition to changes in the immune profile, cancer may be associated with changes at the metabolic level [98]. Cancer cells have the ability to activate and repress different metabolic pathways. Studying these metabolic changes in cancer cells compared to non-cancer cells may reveal potential metabolic vulnerabilities that can be used therapeutically to reduce cancer progression [91]. The first studies of altered metabolism in tumor cells were carried out by the German biochemist, Nobel Prize winner, and physician Otto Heinrich Warburg. He discovered that cancer cells require much more glucose than healthy cells, because they have a highly inefficient cellular metabolism. Unlike healthy cells, they do not metabolize glucose through the mitochondria, where energy is produced, but instead convert it into lactate (lactic acid), which provides much less energy. The Warburg effect is a mechanism that has been proposed as a basis for the hypothesis that dietary interventions such as the ketogenic diet may exert a negative influence on tumor growth. This is on the grounds that, in contrast to healthy cells, tumor cells are unable to utilize ketone bodies effectively due to their acquired metabolic inflexibility and genomic instability, including mitochondrial dysfunction [145, 165, 167, 183]. As ketone bodies are exclusively metabolized in the mitochondria, cancer cells with impaired mitochondrial function are unable to efficiently metabolize them for energy. Although mitochondrial dysfunction provides an explanation for the inability of cancer cells to utilize ketones effectively as a source of energy, the anti-cancer effects of ketones in an in vitro environment where glucose is the primary energy source are not immediately apparent. The potential beneficial effects of this approach are increasingly being discussed in the literature. One such effect is the inhibition of glycolysis, which reduces the main pathway of energy production for cancer cells [175]. Furthermore, an environment characterized by elevated levels of reactive oxygen species (ROS) is conducive to the proliferation of cancer cells. However, even minor alterations in the redox status can exert a significant influence on their growth and survival [142]. In normal cells, but not in cancer cells, ketones have been demonstrated to reduce mitochondrial ROS production and enhance endogenous antioxidant defenses [160]. The metabolism of ketones in healthy cells in the proximity of the tumor may also exert an inhibitory effect on the growth of cancer cells by creating a less favorable redox environment for their survival. Furthermore, ketone bodies are transported into the cell by the monocarboxylate transporters (MCTs), which are also responsible for lactate export. The inhibition of MCT1 activity or lactate export from the cell has been demonstrated to markedly reduce cancer cell growth and survival of cancer cells [33].

Although there has been considerable recent progress in the understanding of the metabolic regulation of cancer cells, it is not yet possible to make clear recommendations on how metabolism can be manipulated to improve responsiveness to ICI [42]. A better understanding of the metabolic regulation of cancer cells is therefore still an active area of research, as is the investigation of suitable methods for targeting the cell metabolism in combination with ICI [111].

Reprogramming of cellular metabolism to enhance cell survival is a characteristic feature of cancer cells. They have the ability to up- and down-regulate intrinsic metabolic pathways in different settings of the primary or metastatic state to adapt to a state of unregulated cell growth [56, 80]. This is achieved by upregulating nutrient uptake and anabolic processes. The main sources of energy are glucose, amino acids, and lipids, with glucose being the primary source of energy. It is derived from ingested carbohydrates or produced de novo by anabolic processes in the liver or kidneys. When glucose is absorbed from the intestine by glucose transporters (GLUT), it can be metabolized intracellularly by glycolysis to pyruvate, 2 ATP and 2 reduction equivalents of NADH + H + . Depending on the oxygen supply, pyruvate is now converted to acetyl-CoA by the pyruvate dehydrogenase (PDH) complex and delivered to the tricarboxylic acid (TCA) cycle. This produces additional reduction equivalents that can contribute to ATP synthesis via the respiratory chain (oxidative phosphorylation). Under anaerobic conditions, such as in hypoxic cells, pyruvate is converted to lactate by lactate dehydrogenase (LDH). The NADH + H + is also oxidized to NAD + and becomes available for a new cycle of glycolysis [18]. In certain rapidly proliferating cells, such as T cells, embryonic stem cells, and cancer cells, high levels of glycolysis and subsequent increased lactate production can also occur independently of oxygen levels [19, 159, 166]. For many years, lactate was considered to be a waste product of glycolysis. However, it has since been identified as an important energy source for cancer cells and as a hallmark of reprogrammed cell metabolism [43, 62, 131]. This has been demonstrated in mice using stable isotopes, which have shown in fasted mice that the incorporation of lactate into intermediates of the TCA cycle is higher than that of glucose in all tissues except the brain [45, 61, 131].

In addition to glucose and lactate, fructose plays a significant role in cellular metabolism. It is not only metabolized by the small intestine and liver, which are the primary sites of fructose uptake, but also by other tissues, including the kidneys, prostate, and bone marrow [16, 66, 81]. A number of studies in mice have indicated that fructose may contribute to the reprogramming of anabolic pathways and thus to cell survival [153]. This ability may be particularly important in solid tumors [12, 23, 171].

Other nutrients, such as amino acids, can also be utilized for anabolic processes. Glutamine is a non-essential amino acid that is either consumed in the diet or synthesized through muscle breakdown and de novo synthesis pathways. Glutamine can be converted to α-ketoglutarate, providing carbon for intermediates of the TCA cycle, which can then be utilized for various anabolic processes, including fatty acid synthesis [32]. In contrast to glutamine, the branched-chain amino acids (leucine, isoleucine, and valine; BCAAs = "branched-chain amino acids") are essential amino acids that must mainly be ingested through food. Preclinical studies in mice have demonstrated that these amino acids are taken up by many tissues and tumors to be subsequently incorporated into newly synthesized proteins or oxidized as fuel [100, 114].

Fatty acids represent another significant source of energy. They possess a high energy density and can be ingested with food and stored in the body in the form of triglycerides. When food intake is low, mammals are able to break down these triglycerides into fatty acids and glycerol. The fatty acids then undergo beta-oxidation, which produces acetyl-CoA and the reduction equivalents FADH2 and NADH + . The resulting acetyl-CoA is transferred to TCA cycle, where it can contribute to the production ATP via the respiratory chain. In the liver, the oxidation of fatty acids produces ketone bodies, which are released into the blood and fed into the TCA cycle in certain tissues [61]. This process is upregulated in rapidly proliferating tissue, such as tumor tissue [100, 149].

Understanding the well-characterized metabolic changes in cancer cells reveals that other cell populations embedded in the tumor microenvironment (TME), such as T cells, macrophages, natural killer cells (NKs), myeloid-derived suppressor cells (MDSCs), and neutrophils from the innate immune system, could be profoundly influenced due to these nutrient limitations [159]. This influence may lead infiltrating immune cells to undergo metabolic adaptations associated with tolerant phenotypes. For instance, T cells, which are the primary targets of ICIs, compete with the rapidly proliferating cancer cells for nutrients to maintain a sufficient anti-tumor response [31, 124]. It is well established that metabolic alterations play a pivotal role in T-cell differentiation, effector function, and the induction of memory T cells. Exhausted T cells exhibit metabolic insufficiency, which can adversely affect immunity and result in a suboptimal response to ICIs [75]. Consequently, the metabolic microenvironment of the tumor may be considered an immunosuppressive milieu that must be overcome. Ultimately, these metabolic changes in immune cells can compromise the efficacy of the anti-tumor immune response upon ICI therapy [125, 150].

### Cardiac metabolism

The heart is an organ with exceptionally high energy requirements. To maintain the heart's contractile function, it is necessary for the heart to produce large amounts of ATP on a constant basis. To achieve this, the heart is capable of metabolizing a variety of fuels, including fatty acids, glucose, lactate, ketones, pyruvate, and amino acids. This is primarily and almost entirely achieved through oxidative phosphorylation in the mitochondria. The process is considerably more efficient in generating ATP than glycolysis [22].

In general, the primary function of cardiac metabolism is to furnish the energy necessary for the heart’s own functions and to ensure that the body’s peripheral organs are supplied with an adequate supply of oxygenated blood. Under a healthy cardiac metabolism, the majority of energy required to fuel the cardiomyocytes is provided via ATP. The remaining third is required for maintaining cellular homeostasis and intracellular electrolyte gradients for electrical stability. However, the ATP storage can be depleted within a short period of time, since the human heart must contract incessantly.

As previously stated, continuous ATP synthesis is vital for maintaining cardiac function. Fatty acids represent as the primary source of ATP production in cardiomyocytes [88]. A comprehensive examination of the utilization of fuel by the human heart revealed that 57% of cardiac ATP produced by the heart is derived from free fatty acids (FFA). The remaining ATP production is attributed to 28% from lipoprotein-derived fatty acids (LpFA), 6.4% from ketones, 4.6% from amino acids, and 2.8% from lactate [108]. The powerhouse of the cell is the mitochondrion, which generates more than 95% of the ATP used by the myocardium. This emphasizes the significance of a constant substrate supply to the mitochondrion. Nevertheless, it has been shown that metabolites generated by both ATP-producing and non-ATP-producing pathways can have a significant impact on adequate cell function [76].

The human heart is capable of utilizing all classes of energy substrates for the synthesis of ATP within the mitochondria [112]. In a healthy cardiac metabolism, fatty acyl-CoA and pyruvate constitute the main substrate for energy production. Long-chain acyl-CoA is converted into long-chain acylcarnitine by carnitine-palmitoyl transferase I (CPT1), which is located on the outer mitochondrial membrane. Subsequently, the long-chain acylcarnitine is transported into the mitochondrion. Pyruvate is transported into the mitochondrion via the mitochondrial pyruvate carrier (MPC), where it is subsequently oxidized by the pyruvate dehydrogenase (PDH) reaction within the mitochondrial matrix. This reaction yields acetyl-Co A (CoA) which then enters the TCA cycle [109]. Fatty acids have been demonstrated to yield the highest ATP production per 2-carbon unit when compared to other substrates. However, it is also notable that they require the most oxygen for ATP production. Therefore, they are the least efficient energy substrates for the heart when evaluated in terms of the ATP generated per unit of oxygen consumed [87].

The role of ketone bodies as a crucial energy source for the heart is becoming increasingly recognized. Unlike lactate, which must undergo conversion to pyruvate by lactate dehydrogenase prior to entering the mitochondrion, ketone and amino bodies can directly enter the mitochondrion [26, 62, 73, 108]. In contrast, amino acid catabolism results in the production of keto acids, which are subsequently metabolized into acetyl-CoA and succinyl-CoA before entering the TCA cycle. In a healthy heart, the contribution of ketone bodies and amino acids to energy production is likely to be small, given their low availability under physiological conditions [67, 101, 172].

Another essential energy substrate for the heart is glucose, which is converted into ATP through two distinct metabolic processes: glycolysis in the cytoplasm and mitochondrial oxidation of pyruvate, which is derived from glycolysis [87]. Glucose catabolism by glycolysis mainly produces pyruvate for oxidation. However, intermediates of glucose are involved in a variety of additional pathways that do not lead to ATP production. Despite their small fluxes, these pathways are of great biological importance. For example, the hexosamine biosynthetic pathway leads to the production of glucose-6-phosphate. The pentose phosphate pathway (PPP) converts glucose-6-phosphate to nicotinamide adenine dinucleotide phosphate (NADPH) during the oxidative phase [76].

NADPH is crucial for antioxidant defense, as it is required to maintain sufficient levels of reduced glutathione, which is necessary for protection against oxidative stress [174]. 5-Carbon sugars, end products of the PPP during the non-oxidative phase, are also important as ribose-5-phosphate becomes a substrate for nucleotide and nucleic acid synthesis [185].

### Metabolism in cancer and immune cells and their adaptations to ICI

As described above, not all patients benefit equally from treatment with ICIs. The specific mechanisms that lead to ICI response, resistance, or even the occurrence of irAEs remain unclear and are a complex area of ongoing research. Possible mechanisms include the constitution of the TME and the various metabolic changes occurring in immune and cancer cells that may contribute to the development of resistance and/or irAEs [3, 150]. Due to the high metabolic activity of cancer cells and the limited vascularization, cancer and immune cells are forced to compete for nutrients and oxygen [148]. By competing for and consuming essential nutrients or otherwise reducing the metabolic adaptability of tumor-infiltrating immune cells, cancer cells are able to suppress anti-tumor immunity resulting in an immunosuppressive TME [3, 148]. Therefore, knowledge of the exact mechanisms and metabolic adaptations may provide opportunities for new or additional interventions to improve the efficacy of ICI.

In T cells, the main target of ICI therapy, activation is defined as the transformation of resting naïve T cells into effector T cells, and is a process with high nutrient requirements. To meet this increasing energy demand, T cells undergo a predominant metabolic reprogramming from oxidative phosphorylation to aerobic glycolysis [47, 164]. At the same time, mitochondrial function is enhanced initiating a shift from catabolic to anabolic processes [95]. As glycolysis and glutamine metabolism are the main metabolic processes, expression of several key components: The glucose transporter GLUT1/SLC2A, the glutamine transporter ASCT2, and sodium-coupled neutral amino acid transporters (SNATs) were assessed. Additionally, the upregulated expression of glycolytic enzymes including hexokinase 2 (HK2) pyruvate dehydrogenase kinase isoform 1 (PDK1), and lactate dehydrogenase A (LDHA), may indicate increased metabolic activity [15, 110]. These metabolic adaptations should support the effector function of T cells. In the context of the TME of solid tumors, the level of glucose is observed to be low in comparison to other metabolic by-products, such as lactate. To ensure anti-tumor control, effector T cells engage aerobic glycolysis [20, 59]. In the challenging environment of the TME, which is characterized by hypoxic conditions and a lack of glucose and amino acids [119]. These conditions influence metabolic adaptability of CD8 + T cells and the increased oxygen consumption of rapidly proliferating cancer cells. These exhausted CD8 + T cells must adapt to the TME by switching from glycolysis to mitochondrial fatty acid β-oxidation (FAO), down-regulating glutaminolysis, reducing mitochondrial biogenesis, and producing more reactive oxygen species (ROS) [140]] In exhausted T cells, lipid uptake and the expression of metabolism-related enzymes are increased. These include the over-expression of CD36 or acyl-CoA synthetase long-chain family member 4 (ACSL4), which can lead to ferroptosis, an iron-dependent form of non-apoptotic cell death regulated by the accumulation of lipid peroxides [34, 85, 94]. Regarding the cholesterol metabolism of exhausted T cells, cholesterol acyltransferase (ACAT) and 3-hydroxy-3-methylglutaryl coenzyme A reductase (HMGCR) have been reported to be involved [94, 177]. With regard to ICI, the crosstalk between (exhausted) T cells and immune-checkpoint signaling is complicated, but knowledge of these interactions may provide new opportunities to enhance the efficacy of ICI by combining T-cell metabolic targeting and ICI.

Recently, there has been increasing evidence that ICI may affect T-cell metabolism. The CD28-mediated activation of the PI3K/Akt pathway leads to increased glucose uptake and metabolism in T cells, which could be inhibited by PD-1 or CTLA-4 [116, 122, 123].

In addition, the ability of the T cells to take up and utilize branched-chain amino acids and glutamine is impaired, while fatty acid oxidation is maintained by PD-1 signaling [123, 155]. Unlike PD-1, CTLA-4 preserves the metabolic profile of unstimulated T cells by inhibiting glycolytic reprogramming without increasing the rate of fatty acid β-oxidation. This suggests that targeting T-cell metabolism in combination with PD-1 blockade may provide enhanced therapeutic efficacy [123]. Moreover, because mitochondria are key players in cellular metabolism, including glucose, fatty acid, and amino acid metabolism, PD-1 signaling impairs oxidative phosphorylation in T cells by reducing mitochondrial fitness [116, 143].

In addition to the T-cell population, other immune cells found in the TME include neutrophils. They could also influence and contribute to tumor control and growth [65]. On the one hand, neutrophils play an essential role in fighting against cancer cells but on the other hand, they could lead to an immunosuppressive and metastasis-promoting environment by promoting angiogenesis and tumor growth [14]. A key metabolic pathway in this setting is the PPP, which neutrophils use to produce more NADPH and reduce ROS levels under conditions of high oxidative stress levels [11, 170]. However, by increasing PPP-derived NADPH, cancer cells are able to boost their antioxidant defenses, which further promotes their growth. Immunosuppression is often associated with increased ROS production by NGs, which inhibits other immune cells, particularly T cells [133].

Another population of immune cells in the TME are tumor-associated macrophages (TAMs), which are known to be divided into two distinct subpopulations, (i) pro-inflammatory (M1) and (ii) immunosuppressive (M2). Under certain circumstances, they have the ability to switch between their subpopulations. In particular, the hypoxic and nutrient-depleted TME with a low pH milieu represents a significant factor in the differentiation of macrophages toward the M2 phenotype [3]. In addition, the secretion of arginase could lead to an immunosuppressive zone and subsequent inhibition of T-cell activity [13]. These effects could be enhanced by the expression of PD-L1 and PD-1 on the surface of TAMs [38]. In conclusion, TAMs could interfere with anti-PD-1 ICI by reducing the access of cytotoxic T cells to the tumor [126]. Notably, blockade of the PD-L1/PD-1 interaction reverses the phagocytosis of PD-1 + TAMs, leading to a reduction in tumor burden [54]. In addition, the transfer of an anti-PD-1 antibody from CD8 + T cells to TAMs is mediated by Fc/Fcg receptor binding shortly after administration as recently observed in vivo. Inhibition of this binding reduces anti-PD-1 antibody levels in TAMs and prolongs their retention in CD8 + T cells, contributing to tumor regression [4].

In addition to the up-regulation of GLUT1 in T cells, this was also observed in NK cells in the TME. Preclinical data have shown that the expression of GLUT1 in NK cells is increased by the hypoxic environment. This suggests that NK cells also shift their metabolism toward glycolysis [127]. In addition, increased GLUT1 expression has been shown to impair the ability of NK cells to fight the tumor by reducing IFN-γ and granzyme B production [139].

Previous studies have investigated the inhibitory effect of ICI on immune cell metabolism by suppressing glycolysis and promoting fatty acid oxidation and lipolysis [129]. Therefore, a better understanding of the interplay between signaling cascades and metabolic networks opens up the possibility of developing new potential treatment combinations [24]. An alternative approach is to combine ICI with metabolic interventions to improve anti-tumor effects by reducing immunometabolic dysfunction.

As metabolic reprogramming is also a hallmark of cancer progression, we will now focus on metabolic changes in cancer metabolism and its adaptation to ICIs [44]. Primary or acquired resistance to ICIs could be caused by both intrinsic and extrinsic factors. Some of the intrinsic factors include intrinsic molecular features of the tumor that could affect the ability of the immune system to generate an adequate anti-tumor response. These circumstances could be exacerbated by the compromised TME due to a reduction in the quality and quantity of immune cells. For example, major histocompatibility complex (MHC) downregulation leads to changes in interferon (INF)-y signaling and dysregulation of tumorigenic pathways, including beta-catenin, p53, and RAS/RAF/MAPK signaling. In addition, expression of PD-L1 leads to T-cell exhaustion following ligand binding with its corresponding ligand PD-1 [84, 120].

As in T cells, the preferred metabolic pathway of cancer cells is glycolysis to meet the high energy demands, which is known to be a major factor in the development of ICI resistance in melanoma patients [17, 25, 97, 179]. This effect was recently demonstrated in a study by Cascone and colleagues. Glycolysis-related genes, such as GAPDH, ALDOA, and ENO2, were negatively correlated with T-cell infiltration in tumor samples from melanoma patients. This suggests that tumor glycolysis may inhibit T-cell-mediated apoptosis in melanoma cells [17]. As mentioned above, the up-regulation of glycolysis is accompanied by an increased over-expression and dysregulation of specific glucose transporters (GLUTs) and hexokinases (HK), which is strongly associated with human cancers [107, 154].

Ongoing studies such as MEL-META and PHENOMENAL are attempting to gain deeper insights into the exact metabolism of cancer cells and its impact on ICI therapy outcome. For example, the MEL-META study is investigating the metabolism of melanoma cells undergoing immunotherapy in relation to the development of resistance to therapy and the metabolic adaptations are associated with this (NCT05307289). PHENOMENAL, an observational study led by Julie Charles, dermatologist at the University Hospital of Grenoble, is investigating the phenotypic, functional, metabolic, and transcriptomic profiling of circulating immune cells to uncover response signatures to ICI in melanoma patients (NCT06154668). Influencing treatment response through direct dietary intervention is currently being investigated in the BREAKFAST-2 study. This multicenter, open-label, two-arm, comparative, randomized phase II trial is investigating whether metabolic interventions consisting of cycles of fasting could improve the anti-cancer activity of standard preoperative chemo-immunotherapy in patients with localized invasive triple-negative breast cancer (NCT05763992) [161].

### Changes in cardiac metabolism

The mechanisms underlying ICI-associated cardiovascular toxicities remain incompletely elucidated. In view of the substantial interest in this field, a considerable body of preclinical and clinical research has already been undertaken. The results of these studies indicate that the following aspects are of great importance: T-cell activation, the mechanisms and functions of ICIs, and the causes and processes of immune-related cardiotoxicity [162].

With particular regard to myocardial metabolism and its adaptations during ICI, it is essential to elucidate the underlying mechanisms associated with cardiovascular complications and to develop strategies for their prevention and management. It is well known that metabolic remodeling is not only a common feature of cancer but also of heart failure. Both cancer cells and cardiomyocytes undergo extensive metabolic reprogramming in response to stress and physiological changes [30]. Overall, glucose is the major substrate required to provide sufficient ATP and metabolic intermediates essential for the synthesis of macromolecules such as fatty acids and nucleotides.

**Fig. 2:**
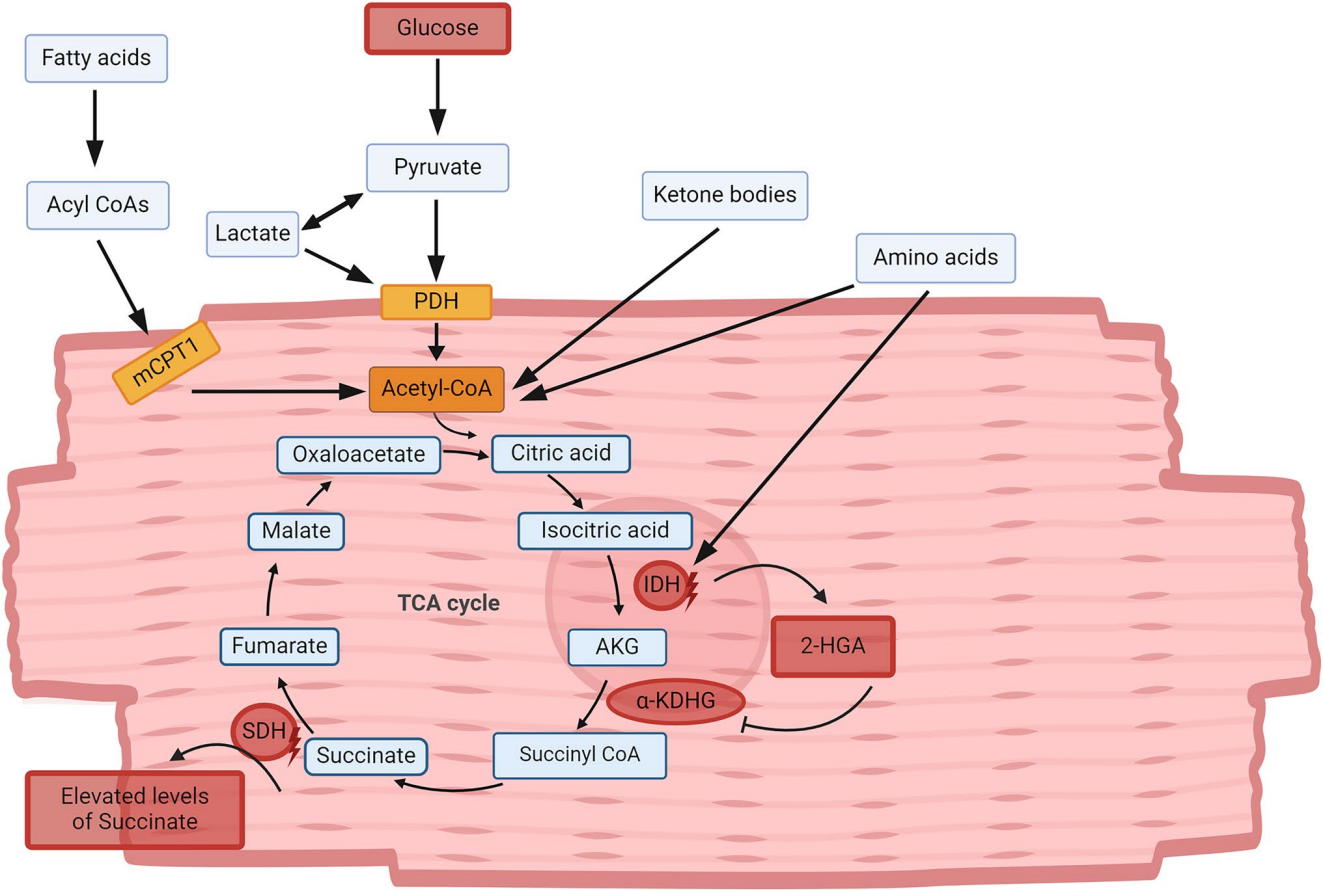
Metabolic pathways in cardiomyocytes and alterations caused by cancer. In healthy myocardium, energy-yielding substrates are converted to Acetyl-CoA, which subsequently enters the TCA cycle. The red boxes indicate alterations in cardiac metabolism under pathological conditions. In diseased cardiomyocytes, glucose becomes the primary energy source. Mutations in IDH1 and IDH2 disrupt ATP production in diseases like acute myeloid leukemia (AML), suggesting that they could also contribute to cardiomyopathy. Additionally, biallelic loss-of-function variants in genes encoding succinate dehydrogenase (SDH) subunits lead to succinate accumulation, which is associated with dilated cardiomyopathy (DCM), cardiovascular disease (CVD), and ischemia–reperfusion injury. *2-HGA* 2-Hydroxyglutarate, *CVD* Cardiovascular Disease, *CoA *Coenzyme A, *DCM* Dilated Cardiomyopathy, *IDH* Isocitrate Dehydrogenase, *mCPT1* Muscle Form of Carnitine Palmitoyl Transferase, *PDH *Pyruvate Dehydrogenase, *SDH *Succinate Dehydrogenase, *α-KDHG* α-Ketoglutarate Dehydrogenase. Figure created with Biorender.com

Healthy cardiomyocytes primarily use fatty acids for energy production in the form of ATP [2]. However, substrate preference can be altered under pathological conditions [64]. An overview about the metabolic pathways in cardiomyocytes and alterations caused by cancer is shown in Fig. 2. In HFrEF, the myocardium undergoes substantial structural damage which results to a decline in mitochondrial function, and consequently, an impaired capacity to oxidize fatty acids. Therefore, the human heart modifies its energy substrate preference from fatty acid oxidation to glucose oxidation to maintain energy production [22, 83]. In contrast, HFpEF is characterized by diastolic dysfunction and systemic inflammation, which results in a distinct metabolic profile in comparison to HFrEF. Furthermore, HFpEF is frequently linked to metabolic disorders, such as obesity, hypertension, and diabetes. Additionally, there is evidence that fatty acid oxidation may be elevated in individuals with HFpEF. This is primarily attributable to the coexistence of insulin resistance, elevated levels of circulating free fatty acids (FFAs) in patients with obesity and diabetes, and alterations in mitochondrial metabolism to meet energy demands. Furthermore, glucose oxidation can be diminished in patients with HFpEF due to a reduction in the ability to take up glucose in insulin-resistant conditions. Additionally, evidence suggest that GLUT4 expression and function are decreased in HFpEF patients [104].

Changes in metabolism remodel metabolic fluxes and contribute to biomass synthesis, which subsequently lead to left-ventricular hypertrophy growth and protein O-GlcNAcylation. This process may lead to calcium mishandling and subsequent cardiac dysfunction [134, 158]. In conclusion, metabolic remodeling in both cancer cells and cardiomyocytes aims at a shift toward anabolic metabolism to enable cell proliferation and hypertrophy, respectively.

Systemic metabolic changes caused by cancer cells have the potential to affect not only the TME, but also to cause changes in several other tissues, including the myocardium [1, 71]. Malignancies due to mutations in the isocitrate dehydrogenase (IDH1/2) gene serve as a prominent example of how mutations that primarily cause cancer can have cardiovascular consequences. Physiologically, IDH1 and IDH2 encode cytosolic and mitochondrial isocitrate dehydrogenase, respectively. Somatic mutations in these genes cause excessive accumulation of the oncometabolite D-2-hydroxyglutarate (D2-HG) in cancer cells. D2-HG inhibits α-ketoglutarate-dependent dioxygenases, which favors tumor growth and proliferation [27, 89]. D2-HG has also been shown to have the potential to affect cardiomyocytes by affecting oxidative metabolism via inhibition of α-ketoglutarate-dependent dioxygenase, ultimately inhibiting ATP production and contractile function [49, 72]. In addition, the production and release of D2-HG is directly related to the development of cardiomyopathy [1]. Kattih et al. additionally demonstrated that mutations in IDH1 and IDH2 are associated with an elevated risk of cardiac dysfunction in patients with acute myeloid leukemia (AML) relative to those with wild-type IDH1 and IDH2. They suggest that the assessment of IDH1 and IDH2 mutations may assist in the identification of AML patients who are at an elevated risk of developing cardiovascular toxicity during the course of potentially cardiotoxic therapies [74]. Further evidence suggests that IDH mutations are associated with a reduction in ATP synthesis and exert a direct influence on energetic metabolism. In a mouse model with homozygous IDH mutations, there was a notable decrease in mitochondrial maximal respiration [46, 121].

From a broader perspective, 2-hydroxyglutaric acidurias are a heterogeneous group of genetic disorders that all lead to the accumulation of either D2-HG or 1–2-hydroxyglutarate (L2-HG). The accumulation of metabolites is caused by mutations in genes, such as D2HGDH, L2HGDH, IDH2, or SLC25A1. The mitochondrial D2-HG and L2-HG dehydrogenases (genes D2HGDH and L2HGDH) physiologically convert D2-HG and L2-HG to α-ketoglutarate. Mutations in D2HGDH or L2HGDH can therefore cause an accumulation of their respective substrates, resulting in either dilated or hypertrophic cardiomyopathy in children and adults [71, 77]. In adult mice, accumulation of plasma D2-HG and L2-HG by induction of variant idh2 expression caused dilated cardiomyopathy and muscular dystrophy. The myocardium of these mice contained fewer mitochondria and more glycogen than the myocardium of control mice [1].

Baysal and colleagues found that biallelic loss-of-function variants in genes encoding subunits of succinate dehydrogenase (SDH) can cause mitochondrial respiratory chain disorders [7]. Subunits of SDH are encoded by SDHA, SDHB, SDHC, and SDHD. Mutations affecting at least one of these genes cause a variety of tumors, such as pheochromocytoma, paraganglioma, gastrointestinal stomal tumors, and papillary renal cell carcinoma [5, 53, 156]. SDH forms complex II of the mitochondrial electron transport chain and subsequently enables the oxidation of succinate to fumarate. Therefore, mutations in genes encoding subunits of the SDH complex lead to the accumulation of succinate. Early research suggests that these changes may lead to dilated cardiomyopathy with impaired left-ventricular function [28, 132, 136]. In addition, elevated plasma succinate levels are associated with cardiovascular disease and ischemia–reperfusion injury [118, 128].

It has been shown that these changes of mitochondrial function can directly disrupt cellular energy homeostasis through dysregulation of endolysosomal and T-cell function, which in turn can culminate in ICI-related cardiotoxicity [141].

In a recent report, Johnson and colleagues detailed two cases of fatal myocarditis accompanied by myositis in patients undergoing immunotherapy with immune-checkpoint inhibitors (ICIs). It is hypothesized that these adverse effects are a consequence of aberrant activation of autoreactive T cells, which serves to illustrate the potential for severe cardiac complications, particularly in the context of combination therapies such as nivolumab and ipilimumab [68]. A number of mouse models have indicated that the depletion or absence of PD-L1 may result in the development of dilated cardiomyopathy or T-cell-dependent myocarditis, potentially due to a reduction in T-cell activity within the heart [36, 48, 55, 68, 90, 151, 168].

While overall data on the influence of ICI on myocardial energy metabolism in cancer are limited, several key proteins that influence mitochondrial function and are dysregulated by ICI have been identified, including mTOR, GSK3ß, PTPN11, and MFN2 [181]. As mTOR is an important regulator of fatty acid metabolism, glycolysis, and oxidative stress response, alterations in mTOR can lead to functional and structural impairment of myocardial function [163, 176]. ICI-related cardiotoxicity is known to be largely driven by hyperinflammatory processes in the myocardium. A key mediator of intracellular inflammation is the NLRP3 inflammasome, which in turn is being modified by GSK-3ß. GSK-3ß is not only directly involved in myocardial inflammation but is also a key regulator of glycogen metabolism and downstream insulin signaling [157, 182]. Its involvement in glycogen metabolism is an additional mediator of ICI-induced cardiotoxicity [105]. The mitochondrial fusion protein (MFN2) is involved in mitochondrial homeostasis and can induce mitochondrial oxidative stress, making the myocardium susceptible to further damage by reactive oxygen species [60]. Overall, the mitochondrial respiratory chain has been shown to be slowed with reduced activity of critical enzymes, such as NADH dehydrogenase, ATP synthase, and FAD synthase. Further studies of how ICI-induced cardiotoxicity alters cardiac function showed that after ICI, overall myocardial lipid metabolism was altered to the extent that lipolysis, a major myocardial energy source, was reduced. In particular, β-oxidation substrates were accumulated intracellularly and the carnitine/acylcarnitine shuttle was attenuated. Reduced fatty acid metabolism, slowed myocardial respiratory chain activity, and altered glycogen metabolism are the hallmarks of impaired myocardial energetic homeostasis under ICI therapy, leaving the myocardium vulnerable to further damage from dysregulated inflammation and reactive oxygen species [102].

Regarding cardiovascular complications of cancer therapy, a prospective, randomized, controlled, multicenter phase-III clinical trial is evaluating early specialized cardiovascular intervention based on impedance cardiography in patients with locally advanced NSCLC receiving concurrent chemoradiation and immunotherapy (NCT04980716)[63]. Such studies may provide a further understanding of the occurrence of irAEs such as cardiovascular complications.

In summary, the immune-checkpoint signaling pathways are implicated in a number of physiological and pathological processes within the heart. In particular, the following three mechanisms appear to be of particular importance in the context of the occurrence of ICI-associated cardiovascular toxicities: (1) Impairment of CTLA-4, PD-L1, PD-1, and LAG-3 signaling by ICIs, thereby lowering the threshold for T-cell activation, and (2) the presence of shared antigens between tumor and heart muscle can lead to the infiltration of activated T cells, macrophages, and monocytes into the heart. This infiltration can trigger various muscle disorders. (3) An increased expression and accumulation of cytokines associated with inflammation caused via ICI therapy triggering the injury [86, 180] (see Table 1).

**Table 1:** Metabolic adaptations in cancer and immune cells: implications for cardiomyocyte response to ICI

Cell type	Key metabolic pathways	Adaptations to cancer	Adaptations to ICI	Implications
Cancer cells	Glycolysis (Warburg effect)—due to (i) high requirement of glucose and (ii) an inefficient cellular metabolism → Altered nutrient utilization	–	Upregulated alternative splicing of pyruvate kinase muscle (PKM2) from PKM pre-mRNA → aerobic glycolysis	Potential for restricting rapid growth and proliferation
	Glutaminolysis—glutamine to α-ketoglutarate providing carbon for intermediates of the TCA cycle → Resistance to metabolic stress	–	Current research topic	Potential for immune evasion
	Increased lipid metabolism—providing intermediates and precursors to the TCA cycle → Enhanced survival under immune pressure	–	Current research topic	Contributes to tumor microenvironment
Cardiomyocytes	Fatty acid oxidation—fatty acids serving as the primary source of ATP production	Shift to glycolysis during stress	Permanently reduction of the myocardial lipid metabolism and altered glycogen metabolism	Risk of heart failure under high inflammatory states
	Glucose utilization—generating ATP through both glycolysis and mitochondrial oxidation of pyruvate	Increased mitochondrial biogenesis	May be altered by ICI	Increased vulnerability to further damage
	Metabolic flexibility	Altered substrate use to cope with energy needs	Current research topic	Essential for resilience and recovery
T cells	Naïve T cells: Oxidative phosphorylation	Shift to aerobic glycolysis Enhanced mitochondrial function	CTLA-4 preserves metabolic profile by inhibiting glycolytic reprogramming without increasing fatty acid β-oxidation	Targeting T-cell metabolism in combination with PD-1 blockade may provide enhanced therapeutic efficacy
	Activated T cells: Aerobic glycolysis	Increased expression of several key components and glucolytic enzymes Exhausted CD8 + T cells: Shift from glycolysis to mitochondrial fatty acid β-oxidation (FAO) Down-regulation of glutaminolysis Reduction of mitochondrial biogenesis Production of reactive oxygen species (ROS) Increased lipid uptake Occurrence of ferroptosis	PD-1: Impairment of amino acid and glutamine metabolism Alteration of metabolic reprogramming by inhibiting glycolysis and promoting lipolysis and fatty acid oxidation Impairment of oxidative phosphorylation	Impacts immune efficacy in tumor clearance
	Memory T cells: Reliance on oxidative phosphorylation	Enhanced longevity and function	Current research topic	Supports sustained immune response
NK cells	Primarily glycolysis for rapid energy production	Metabolic reprogramming upon activation	Current research topic	Enhanced cytotoxicity against tumor cells
	Fatty acid oxidation during rest	Increased reliance on oxidative metabolism for sustained activity	Current research topic	Contributes to anti-tumor immunity
TAMs	Primarily rely on glycolysis in the tumor microenvironment	Shift toward anti-inflammatory M2 phenotype	Current research topic	Can influence tumor growth and immune suppression
	Can switch to oxidative phosphorylation under pro- inflammatory signals	Adapt metabolic pathways to support tumor progression	Current research topic	Role in immune evasion and metastasis
	Secretion of arginase could lead to an immunosuppressive zone	Inhibition of T-cell activity Increased expression of PD-L1 and PD-1 on the surface of TAMs	Blockage of the PD-L1/PD-1 interaction reverses the phagocytosis of PD-1 + TAMs	May lead to a reduction in tumor burden

Highlighting the metabolic adaptations in healthy conditions, alongside those observed in cancer and in response to ICIs

## Conclusion

In conclusion, the interaction between ICIs and metabolic pathways is crucial for understanding the complex responses to cancer therapies and potential adverse effects, particularly in relation to cardiotoxicity. As previously indicated, the mechanisms underlying differential patient responses to ICIs—including resistance and the development of irAEs—are complex and are significantly influenced by the TME as well as metabolic alterations in both cancer and immune cells. A more comprehensive grasp of these metabolic adaptations, particularly in T cells and other immune cell populations, may facilitate the development of novel therapeutic strategies that integrate ICIs with metabolic interventions to enhance treatment efficacy.

In the future, the incorporation of metabolic profiling into patient stratification will facilitate the identification of individuals who are most likely to benefit from targeted therapies, thereby enabling the implementation of more personalized treatment approaches. Furthermore, the metabolic alterations observed in cardiomyocytes during the development of cancer and ICI therapy highlight the complexity of identifying metabolic vulnerabilities. It suggests that a singular target is not always the most effective approach, and that a combination of different therapies is often necessary. While the use of ICIs has yielded significant benefits in the field of oncology, it has also brought to light a number of associated risks, including cardiovascular complications. This underscores the continued necessity for targeted strategies aimed at preventing and managing these adverse effects.

It is imperative that further preclinical and clinical studies be conducted, rather than relying solely on animal models, to effectively translate these findings into clinical practice. Further research in the field of metabolism has the potential to elucidate the complex relationships between metabolic pathways, immune responses, and cardiovascular health in the context of cancer treatment. Ultimately, the goal of this research is to optimize therapy and reduce risks for patients.

## References

[1] Akbay EA, Moslehi J, Christensen CL, Saha S, Tchaicha JH, Ramkissoon SH, Stewart KM, Carretero J, Kikuchi E, Zhang H, Cohoon TJ, Murray S, Liu W, Uno K, Fisch S, Jones K, Gurumurthy S, Gliser C, Choe S, Keenan M, Son J, Stanley I, Losman JA, Padera R, Bronson RT, Asara JM, Abdel-Wahab O, Amrein PC, Fathi AT, Danial NN, Kimmelman AC, Kung AL, Ligon KL, Yen KE, Kaelin WG Jr, Bardeesy N, Wong KK (2014) D-2-hydroxyglutarate produced by mutant IDH2 causes cardiomyopathy and neurodegeneration in mice. Genes Dev 28:479–490. 10.1101/gad.231233.113 24589777 10.1101/gad.231233.113 PMC3950345

[2] Allard MF, Schonekess BO, Henning SL, English DR, Lopaschuk GD (1994) Contribution of oxidative metabolism and glycolysis to ATP production in hypertrophied hearts. Am J Physiol 267:H742-750. 10.1152/ajpheart.1994.267.2.H742 8067430 10.1152/ajpheart.1994.267.2.H742

[3] Andrejeva G, Rathmell JC (2017) Similarities and distinctions of cancer and immune metabolism in inflammation and tumors. Cell Metab 26:49–70. 10.1016/j.cmet.2017.06.004 28683294 10.1016/j.cmet.2017.06.004 PMC5555084

[4] Arlauckas SP, Garris CS, Kohler RH, Kitaoka M, Cuccarese MF, Yang KS, Miller MA, Carlson JC, Freeman GJ, Anthony RM, Weissleder R, Pittet MJ (2017) In vivo imaging reveals a tumor-associated macrophage-mediated resistance pathway in anti-PD-1 therapy. Sci Transl Med. 10.1126/scitranslmed.aal3604 28490665 10.1126/scitranslmed.aal3604 PMC5734617

[5] Astuti D, Latif F, Dallol A, Dahia PL, Douglas F, George E, Skoldberg F, Husebye ES, Eng C, Maher ER (2001) Gene mutations in the succinate dehydrogenase subunit SDHB cause susceptibility to familial pheochromocytoma and to familial paraganglioma. Am J Hum Genet 69:49–54. 10.1086/321282 11404820 10.1086/321282 PMC1226047

[6] Baik AH, Oluwole OO, Johnson DB, Shah N, Salem JE, Tsai KK, Moslehi JJ (2021) Mechanisms of cardiovascular toxicities associated with immunotherapies. Circ Res 128:1780–1801. 10.1161/CIRCRESAHA.120.315894 33934609 10.1161/CIRCRESAHA.120.315894 PMC8159878

[7] Baysal BE, Ferrell RE, Willett-Brozick JE, Lawrence EC, Myssiorek D, Bosch A, van der Mey A, Taschner PE, Rubinstein WS, Myers EN, Richard CW 3rd, Cornelisse CJ, Devilee P, Devlin B (2000) Mutations in SDHD, a mitochondrial complex II gene, in hereditary paraganglioma. Science 287:848–851. 10.1126/science.287.5454.848 10657297 10.1126/science.287.5454.848

[8] Beaver JA, Pazdur R (2022) The wild west of checkpoint inhibitor development. N Engl J Med 386:1297–1301. 10.1056/NEJMp2116863 34910860 10.1056/NEJMp2116863

[9] Bertrand A, Kostine M, Barnetche T, Truchetet ME, Schaeverbeke T (2015) Immune related adverse events associated with anti-CTLA-4 antibodies: systematic review and meta-analysis. BMC Med 13:211. 10.1186/s12916-015-0455-8 26337719 10.1186/s12916-015-0455-8 PMC4559965

[10] Bockstahler M, Fischer A, Goetzke CC, Neumaier HL, Sauter M, Kespohl M, Muller AM, Meckes C, Salbach C, Schenk M, Heuser A, Landmesser U, Weiner J, Meder B, Lehmann L, Kratzer A, Klingel K, Katus HA, Kaya Z, Beling A (2020) Heart-specific immune responses in an animal model of autoimmune-related myocarditis mitigated by an immunoproteasome inhibitor and genetic ablation. Circulation 141:1885–1902. 10.1161/CIRCULATIONAHA.119.043171 32160764 10.1161/CIRCULATIONAHA.119.043171

[11] Britt EC, Lika J, Giese MA, Schoen TJ, Seim GL, Huang Z, Lee PY, Huttenlocher A, Fan J (2022) Switching to the cyclic pentose phosphate pathway powers the oxidative burst in activated neutrophils. Nat Metab 4:389–403. 10.1038/s42255-022-00550-8 35347316 10.1038/s42255-022-00550-8 PMC8964420

[12] Bu P, Chen KY, Xiang K, Johnson C, Crown SB, Rakhilin N, Ai Y, Wang L, Xi R, Astapova I, Han Y, Li J, Barth BB, Lu M, Gao Z, Mines R, Zhang L, Herman M, Hsu D, Zhang GF, Shen X (2018) Aldolase B-mediated fructose metabolism drives metabolic reprogramming of colon cancer liver metastasis. Cell Metab. 10.1016/j.cmet.2018.04.003 29706565 10.1016/j.cmet.2018.04.003 PMC5990465

[13] Buscher K, Ehinger E, Gupta P, Pramod AB, Wolf D, Tweet G, Pan C, Mills CD, Lusis AJ, Ley K (2017) Natural variation of macrophage activation as disease-relevant phenotype predictive of inflammation and cancer survival. Nat Commun 8:16041. 10.1038/ncomms16041 28737175 10.1038/ncomms16041 PMC5527282

[14] Capone M, Giannarelli D, Mallardo D, Madonna G, Festino L, Grimaldi AM, Vanella V, Simeone E, Paone M, Palmieri G, Cavalcanti E, Caraco C, Ascierto PA (2018) Baseline neutrophil-to-lymphocyte ratio (NLR) and derived NLR could predict overall survival in patients with advanced melanoma treated with nivolumab. J Immunother Cancer 6:74. 10.1186/s40425-018-0383-1 30012216 10.1186/s40425-018-0383-1 PMC6048712

[15] Carr EL, Kelman A, Wu GS, Gopaul R, Senkevitch E, Aghvanyan A, Turay AM, Frauwirth KA (2010) Glutamine uptake and metabolism are coordinately regulated by ERK/MAPK during T lymphocyte activation. J Immunol 185:1037–1044. 10.4049/jimmunol.0903586 20554958 10.4049/jimmunol.0903586 PMC2897897

[16] Carreno D, Corro N, Torres-Estay V, Veliz LP, Jaimovich R, Cisternas P, San Francisco IF, Sotomayor PC, Tanasova M, Inestrosa NC, Godoy AS (2019) Fructose and prostate cancer: toward an integrated view of cancer cell metabolism. Prostate Cancer Prostatic Dis 22:49–58. 10.1038/s41391-018-0072-7 30104655 10.1038/s41391-018-0072-7

[17] Cascone T, McKenzie JA, Mbofung RM, Punt S, Wang Z, Xu C, Williams LJ, Wang Z, Bristow CA, Carugo A, Peoples MD, Li L, Karpinets T, Huang L, Malu S, Creasy C, Leahey SE, Chen J, Chen Y, Pelicano H, Bernatchez C, Gopal YNV, Heffernan TP, Hu J, Wang J, Amaria RN, Garraway LA, Huang P, Yang P, Wistuba II, Woodman SE, Roszik J, Davis RE, Davies MA, Heymach JV, Hwu P, Peng W (2018) Increased tumor glycolysis characterizes immune resistance to adoptive T cell therapy. Cell Metab. 10.1016/j.cmet.2018.02.024 29628419 10.1016/j.cmet.2018.02.024 PMC5932208

[18] Chandel NS (2021) Glycolysis. Cold Spring Harb Perspect Biol. 10.1101/cshperspect.a040535 33941515 10.1101/cshperspect.a040535 PMC8091952

[19] Chandel NS (2021) Metabolism of proliferating cells. Cold Spring Harb Perspect Biol. 10.1101/cshperspect.a040618 34598925 10.1101/cshperspect.a040618 PMC8485748

[20] Chang CH, Qiu J, O’Sullivan D, Buck MD, Noguchi T, Curtis JD, Chen Q, Gindin M, Gubin MM, van der Windt GJ, Tonc E, Schreiber RD, Pearce EJ, Pearce EL (2015) Metabolic competition in the tumor microenvironment is a driver of cancer progression. Cell 162:1229–1241. 10.1016/j.cell.2015.08.016 26321679 10.1016/j.cell.2015.08.016 PMC4864363

[21] Chen DY, Huang WK, Chien-Chia WuV, Chang WC, Chen JS, Chuang CK, Chu PH (2020) Cardiovascular toxicity of immune checkpoint inhibitors in cancer patients: a review when cardiology meets immuno-oncology. J Formos Med Assoc 119:1461–1475. 10.1016/j.jfma.2019.07.025 31444018 10.1016/j.jfma.2019.07.025

[22] Chen S, Zou Y, Song C, Cao K, Cai K, Wu Y, Zhang Z, Geng D, Sun W, Ouyang N, Zhang N, Li Z, Sun G, Zhang Y, Sun Y, Zhang Y (2023) The role of glycolytic metabolic pathways in cardiovascular disease and potential therapeutic approaches. Basic Res Cardiol 118:48. 10.1007/s00395-023-01018-w 37938421 10.1007/s00395-023-01018-w PMC10632287

[23] Chen WL, Jin X, Wang M, Liu D, Luo Q, Tian H, Cai L, Meng L, Bi R, Wang L, Xie X, Yu G, Li L, Dong C, Cai Q, Jia W, Wei W, Jia L (2020) GLUT5-mediated fructose utilization drives lung cancer growth by stimulating fatty acid synthesis and AMPK/mTORC1 signaling. JCI Insight. 10.1172/jci.insight.131596 32051337 10.1172/jci.insight.131596 PMC7098789

[24] Chuang YM, Tzeng SF, Ho PC, Tsai CH (2024) Immunosurveillance encounters cancer metabolism. EMBO Rep 25:471–488. 10.1038/s44319-023-00038-w 38216787 10.1038/s44319-023-00038-w PMC10897436

[25] Courtnay R, Ngo DC, Malik N, Ververis K, Tortorella SM, Karagiannis TC (2015) Cancer metabolism and the Warburg effect: the role of HIF-1 and PI3K. Mol Biol Rep 42:841–851. 10.1007/s11033-015-3858-x 25689954 10.1007/s11033-015-3858-x

[26] Dai C, Li Q, May HI, Li C, Zhang G, Sharma G, Sherry AD, Malloy CR, Khemtong C, Zhang Y, Deng Y, Gillette TG, Xu J, Scadden DT, Wang ZV (2020) Lactate dehydrogenase a governs cardiac hypertrophic growth in response to hemodynamic stress. Cell Rep. 10.1016/j.celrep.2020.108087 32877669 10.1016/j.celrep.2020.108087 PMC7520916

[27] Dang L, White DW, Gross S, Bennett BD, Bittinger MA, Driggers EM, Fantin VR, Jang HG, Jin S, Keenan MC, Marks KM, Prins RM, Ward PS, Yen KE, Liau LM, Rabinowitz JD, Cantley LC, Thompson CB, Vander Heiden MG, Su SM (2009) Cancer-associated IDH1 mutations produce 2-hydroxyglutarate. Nature 462:739–744. 10.1038/nature08617 19935646 10.1038/nature08617 PMC2818760

[28] Davili Z, Johar S, Hughes C, Kveselis D, Hoo J (2007) Succinate dehydrogenase deficiency associated with dilated cardiomyopathy and ventricular noncompaction. Eur J Pediatr 166:867–870. 10.1007/s00431-006-0310-1 17082968 10.1007/s00431-006-0310-1

[29] de Almeida DVP, Gomes JR, Haddad FJ, Buzaid AC (2018) Immune-mediated pericarditis with pericardial tamponade during nivolumab therapy. J Immunother 41:329–331. 10.1097/CJI.0000000000000217 29461982 10.1097/CJI.0000000000000217

[30] de Boer RA, Hulot JS, Tocchetti CG, Aboumsallem JP, Ameri P, Anker SD, Bauersachs J, Bertero E, Coats AJS, Celutkiene J, Chioncel O, Dodion P, Eschenhagen T, Farmakis D, Bayes-Genis A, Jager D, Jankowska EA, Kitsis RN, Konety SH, Larkin J, Lehmann L, Lenihan DJ, Maack C, Moslehi JJ, Muller OJ, Nowak-Sliwinska P, Piepoli MF, Ponikowski P, Pudil R, Rainer PP, Ruschitzka F, Sawyer D, Seferovic PM, Suter T, Thum T, van der Meer P, Van Laake LW, von Haehling S, Heymans S, Lyon AR, Backs J (2020) Common mechanistic pathways in cancer and heart failure. A scientific roadmap on behalf of the Translational Research Committee of the Heart Failure Association (HFA) of the European Society of Cardiology (ESC). Eur J Heart Fail 22:2272–2289. 10.1002/ejhf.2029 33094495 10.1002/ejhf.2029 PMC7894564

[31] DeBerardinis RJ, Chandel NS (2016) Fundamentals of cancer metabolism. Sci Adv. 10.1126/sciadv.1600200 27386546 10.1126/sciadv.1600200 PMC4928883

[32] DeBerardinis RJ, Mancuso A, Daikhin E, Nissim I, Yudkoff M, Wehrli S, Thompson CB (2007) Beyond aerobic glycolysis: transformed cells can engage in glutamine metabolism that exceeds the requirement for protein and nucleotide synthesis. Proc Natl Acad Sci U S A 104:19345–19350. 10.1073/pnas.0709747104 18032601 10.1073/pnas.0709747104 PMC2148292

[33] Dhup S, Dadhich RK, Porporato PE, Sonveaux P (2012) Multiple biological activities of lactic acid in cancer: influences on tumor growth, angiogenesis and metastasis. Curr Pharm Des 18:1319–1330. 10.2174/138161212799504902 22360558 10.2174/138161212799504902

[34] Dixon SJ, Lemberg KM, Lamprecht MR, Skouta R, Zaitsev EM, Gleason CE, Patel DN, Bauer AJ, Cantley AM, Yang WS, Morrison B 3rd, Stockwell BR (2012) Ferroptosis: an iron-dependent form of nonapoptotic cell death. Cell 149:1060–1072. 10.1016/j.cell.2012.03.042 22632970 10.1016/j.cell.2012.03.042 PMC3367386

[35] Dolladille C, Akroun J, Morice PM, Dompmartin A, Ezine E, Sassier M, Da-Silva A, Plane AF, Legallois D, L’Orphelin JM, Alexandre J (2021) Cardiovascular immunotoxicities associated with immune checkpoint inhibitors: a safety meta-analysis. Eur Heart J 42:4964–4977. 10.1093/eurheartj/ehab618 34529770 10.1093/eurheartj/ehab618

[36] Dong H, Zhu G, Tamada K, Chen L (1999) B7–H1, a third member of the B7 family, co-stimulates T-cell proliferation and interleukin-10 secretion. Nat Med 5:1365–1369. 10.1038/70932 10581077 10.1038/70932

[37] Drobni ZD, Alvi RM, Taron J, Zafar A, Murphy SP, Rambarat PK, Mosarla RC, Lee C, Zlotoff DA, Raghu VK, Hartmann SE, Gilman HK, Gong J, Zubiri L, Sullivan RJ, Reynolds KL, Mayrhofer T, Zhang L, Hoffmann U, Neilan TG (2020) Association between immune checkpoint inhibitors with cardiovascular events and atherosclerotic plaque. Circulation 142:2299–2311. 10.1161/CIRCULATIONAHA.120.049981 33003973 10.1161/CIRCULATIONAHA.120.049981 PMC7736526

[38] Duan Z, Luo Y (2021) Targeting macrophages in cancer immunotherapy. Signal Transduct Target Ther 6:127. 10.1038/s41392-021-00506-6 33767177 10.1038/s41392-021-00506-6 PMC7994399

[39] Efentakis P, Choustoulaki A, Kwiatkowski G, Varela A, Kostopoulos IV, Tsekenis G, Ntanasis-Stathopoulos I, Georgoulis A, Vorgias CE, Gakiopoulou H, Briasoulis A, Davos CH, Kostomitsopoulos N, Tsitsilonis O, Dimopoulos MA, Terpos E, Chlopicki S, Gavriatopoulou M, Andreadou I (2024) Early microvascular coronary endothelial dysfunction precedes pembrolizumab-induced cardiotoxicity. Preventive role of high dose of atorvastatin. Basic Res Cardiol. 10.1007/s00395-024-01046-0 38520533 10.1007/s00395-024-01046-0 PMC11790778

[40] Eigentler TK, Hassel JC, Berking C, Aberle J, Bachmann O, Grunwald V, Kahler KC, Loquai C, Reinmuth N, Steins M, Zimmer L, Sendl A, Gutzmer R (2016) Diagnosis, monitoring and management of immune-related adverse drug reactions of anti-PD-1 antibody therapy. Cancer Treat Rev 45:7–18. 10.1016/j.ctrv.2016.02.003 26922661 10.1016/j.ctrv.2016.02.003

[41] Escudier M, Cautela J, Malissen N, Ancedy Y, Orabona M, Pinto J, Monestier S, Grob JJ, Scemama U, Jacquier A, Lalevee N, Barraud J, Peyrol M, Laine M, Bonello L, Paganelli F, Cohen A, Barlesi F, Ederhy S, Thuny F (2017) Clinical features, management, and outcomes of immune checkpoint inhibitor-related cardiotoxicity. Circulation 136:2085–2087. 10.1161/CIRCULATIONAHA.117.030571 29158217 10.1161/CIRCULATIONAHA.117.030571

[42] Evers TMJ, Hochane M, Tans SJ, Heeren RMA, Semrau S, Nemes P, Mashaghi A (2019) Deciphering metabolic heterogeneity by single-cell analysis. Anal Chem 91:13314–13323. 10.1021/acs.analchem.9b02410 31549807 10.1021/acs.analchem.9b02410 PMC6922888

[43] Faubert B, Li KY, Cai L, Hensley CT, Kim J, Zacharias LG, Yang C, Do QN, Doucette S, Burguete D, Li H, Huet G, Yuan Q, Wigal T, Butt Y, Ni M, Torrealba J, Oliver D, Lenkinski RE, Malloy CR, Wachsmann JW, Young JD, Kernstine K, DeBerardinis RJ (2017) Lactate metabolism in human lung tumors. Cell. 10.1016/j.cell.2017.09.019 28985563 10.1016/j.cell.2017.09.019 PMC5684706

[44] Faubert B, Solmonson A, DeBerardinis RJ (2020) Metabolic reprogramming and cancer progression. Science. 10.1126/science.aaw5473 32273439 10.1126/science.aaw5473 PMC7227780

[45] Faubert B, Tasdogan A, Morrison SJ, Mathews TP, DeBerardinis RJ (2021) Stable isotope tracing to assess tumor metabolism in vivo. Nat Protoc 16:5123–5145. 10.1038/s41596-021-00605-2 34535790 10.1038/s41596-021-00605-2 PMC9274147

[46] Findlay AS, Carter RN, Starbuck B, McKie L, Novakova K, Budd PS, Keighren MA, Marsh JA, Cross SH, Simon MM, Potter PK, Morton NM, Jackson IJ (2018) Mouse Idh3a mutations cause retinal degeneration and reduced mitochondrial function. Dis Model Mech. 10.1242/dmm.036426 30478029 10.1242/dmm.036426 PMC6307916

[47] Frauwirth KA, Riley JL, Harris MH, Parry RV, Rathmell JC, Plas DR, Elstrom RL, June CH, Thompson CB (2002) The CD28 signaling pathway regulates glucose metabolism. Immunity 16:769–777. 10.1016/s1074-7613(02)00323-0 12121659 10.1016/s1074-7613(02)00323-0

[48] Freeman GJ, Long AJ, Iwai Y, Bourque K, Chernova T, Nishimura H, Fitz LJ, Malenkovich N, Okazaki T, Byrne MC, Horton HF, Fouser L, Carter L, Ling V, Bowman MR, Carreno BM, Collins M, Wood CR, Honjo T (2000) Engagement of the PD-1 immunoinhibitory receptor by a novel B7 family member leads to negative regulation of lymphocyte activation. J Exp Med 192:1027–1034. 10.1084/jem.192.7.1027 11015443 10.1084/jem.192.7.1027 PMC2193311

[49] Fu X, Chin RM, Vergnes L, Hwang H, Deng G, Xing Y, Pai MY, Li S, Ta L, Fazlollahi F, Chen C, Prins RM, Teitell MA, Nathanson DA, Lai A, Faull KF, Jiang M, Clarke SG, Cloughesy TF, Graeber TG, Braas D, Christofk HR, Jung ME, Reue K, Huang J (2015) 2-Hydroxyglutarate inhibits ATP synthase and mTOR signaling. Cell Metab 22:508–515. 10.1016/j.cmet.2015.06.009 26190651 10.1016/j.cmet.2015.06.009 PMC4663076

[50] Gergely TG, Drobni ZD, Kallikourdis M, Zhu H, Meijers WC, Neilan TG, Rassaf T, Ferdinandy P, Varga ZV (2024) Immune checkpoints in cardiac physiology and pathology: therapeutic targets for heart failure. Nat Rev Cardiol 21:443–462. 10.1038/s41569-023-00986-9 38279046 10.1038/s41569-023-00986-9

[51] Gergely TG, Drobni ZD, Sayour NV, Ferdinandy P, Varga ZV (2024) Molecular fingerprints of cardiovascular toxicities of immune checkpoint inhibitors. Basic Res Cardiol. 10.1007/s00395-024-01068-8 39023770 10.1007/s00395-024-01068-8 PMC11790702

[52] Ghisoni E, Wicky A, Bouchaab H, Imbimbo M, Delyon J, Gautron Moura B, Gerard CL, Latifyan S, Ozdemir BC, Caikovski M, Pradervand S, Tavazzi E, Gatta R, Marandino L, Valabrega G, Aglietta M, Obeid M, Homicsko K, Mederos Alfonso NN, Zimmermann S, Coukos G, Peters S, Cuendet MA, Di Maio M, Michielin O (2021) Late-onset and long-lasting immune-related adverse events from immune checkpoint-inhibitors: an overlooked aspect in immunotherapy. Eur J Cancer 149:153–164. 10.1016/j.ejca.2021.03.010 33865201 10.1016/j.ejca.2021.03.010

[53] Goncalves J, Moog S, Morin A, Gentric G, Muller S, Morrell AP, Kluckova K, Stewart TJ, Andoniadou CL, Lussey-Lepoutre C, Benit P, Thakker A, Vettore L, Roberts J, Rodriguez R, Mechta-Grigoriou F, Gimenez-Roqueplo AP, Letouze E, Tennant DA, Favier J (2021) Loss of SDHB promotes dysregulated iron homeostasis, oxidative stress, and sensitivity to ascorbate. Cancer Res 81:3480–3494. 10.1158/0008-5472.CAN-20-2936 34127497 10.1158/0008-5472.CAN-20-2936 PMC7616967

[54] Gordon SR, Maute RL, Dulken BW, Hutter G, George BM, McCracken MN, Gupta R, Tsai JM, Sinha R, Corey D, Ring AM, Connolly AJ, Weissman IL (2017) PD-1 expression by tumour-associated macrophages inhibits phagocytosis and tumour immunity. Nature 545:495–499. 10.1038/nature22396 28514441 10.1038/nature22396 PMC5931375

[55] Grabie N, Gotsman I, DaCosta R, Pang H, Stavrakis G, Butte MJ, Keir ME, Freeman GJ, Sharpe AH, Lichtman AH (2007) Endothelial programmed death-1 ligand 1 (PD-L1) regulates CD8+ T-cell mediated injury in the heart. Circulation 116:2062–2071. 10.1161/CIRCULATIONAHA.107.709360 17938288 10.1161/CIRCULATIONAHA.107.709360

[56] Hanahan D, Weinberg RA (2011) Hallmarks of cancer: the next generation. Cell 144:646–674. 10.1016/j.cell.2011.02.013 21376230 10.1016/j.cell.2011.02.013

[57] Hassel JC, Heinzerling L, Aberle J, Bahr O, Eigentler TK, Grimm MO, Grunwald V, Leipe J, Reinmuth N, Tietze JK, Trojan J, Zimmer L, Gutzmer R (2017) Combined immune checkpoint blockade (anti-PD-1/anti-CTLA-4): evaluation and management of adverse drug reactions. Cancer Treat Rev 57:36–49. 10.1016/j.ctrv.2017.05.003 28550712 10.1016/j.ctrv.2017.05.003

[58] Heinzerling L, Goldinger SM (2017) A review of serious adverse effects under treatment with checkpoint inhibitors. Curr Opin Oncol 29:136–144. 10.1097/CCO.0000000000000358 28059853 10.1097/CCO.0000000000000358

[59] Ho PC, Bihuniak JD, Macintyre AN, Staron M, Liu X, Amezquita R, Tsui YC, Cui G, Micevic G, Perales JC, Kleinstein SH, Abel ED, Insogna KL, Feske S, Locasale JW, Bosenberg MW, Rathmell JC, Kaech SM (2015) Phosphoenolpyruvate is a metabolic checkpoint of anti-tumor T cell responses. Cell 162:1217–1228. 10.1016/j.cell.2015.08.012 26321681 10.1016/j.cell.2015.08.012 PMC4567953

[60] Homme RP, George AK, Singh M, Smolenkova I, Zheng Y, Pushpakumar S, Tyagi SC (2021) Mechanism of blood-heart-barrier leakage: implications for COVID-19 induced cardiovascular injury. Int J Mol Sci. 10.3390/ijms222413546 34948342 10.3390/ijms222413546 PMC8706694

[61] Hui S, Cowan AJ, Zeng X, Yang L, TeSlaa T, Li X, Bartman C, Zhang Z, Jang C, Wang L, Lu W, Rojas J, Baur J, Rabinowitz JD (2020) Quantitative fluxomics of circulating metabolites. Cell Metab. 10.1016/j.cmet.2020.07.013 32791100 10.1016/j.cmet.2020.07.013 PMC7544659

[62] Hui S, Ghergurovich JM, Morscher RJ, Jang C, Teng X, Lu W, Esparza LA, Reya T, Le Z, Yanxiang Guo J, White E, Rabinowitz JD (2017) Glucose feeds the TCA cycle via circulating lactate. Nature 551:115–118. 10.1038/nature24057 29045397 10.1038/nature24057 PMC5898814

[63] Inci TG, Acar S, Turgut-Balik D (2024) Nonsmall-cell lung cancer treatment: current status of drug repurposing and nanoparticle-based drug delivery systems. Turk J Biol 48:112–132. 10.55730/1300-0152.2687 39051063 10.55730/1300-0152.2687 PMC11265851

[64] Jabs M, Rose AJ, Lehmann LH, Taylor J, Moll I, Sijmonsma TP, Herberich SE, Sauer SW, Poschet G, Federico G, Mogler C, Weis EM, Augustin HG, Yan M, Gretz N, Schmid RM, Adams RH, Grone HJ, Hell R, Okun JG, Backs J, Nawroth PP, Herzig S, Fischer A (2018) Inhibition of endothelial notch signaling impairs fatty acid transport and leads to metabolic and vascular remodeling of the adult heart. Circulation 137:2592–2608. 10.1161/CIRCULATIONAHA.117.029733 29353241 10.1161/CIRCULATIONAHA.117.029733

[65] Jaillon S, Ponzetta A, Di Mitri D, Santoni A, Bonecchi R, Mantovani A (2020) Neutrophil diversity and plasticity in tumour progression and therapy. Nat Rev Cancer 20:485–503. 10.1038/s41568-020-0281-y 32694624 10.1038/s41568-020-0281-y

[66] Jang C, Hui S, Lu W, Cowan AJ, Morscher RJ, Lee G, Liu W, Tesz GJ, Birnbaum MJ, Rabinowitz JD (2018) The small intestine converts dietary fructose into glucose and organic acids. Cell Metab. 10.1016/j.cmet.2017.12.016 29414685 10.1016/j.cmet.2017.12.016 PMC6032988

[67] Jeffrey FM, Diczku V, Sherry AD, Malloy CR (1995) Substrate selection in the isolated working rat heart: effects of reperfusion, afterload, and concentration. Basic Res Cardiol 90:388–396. 10.1007/BF00788500 8585860 10.1007/BF00788500

[68] Johnson DB, Balko JM, Compton ML, Chalkias S, Gorham J, Xu Y, Hicks M, Puzanov I, Alexander MR, Bloomer TL, Becker JR, Slosky DA, Phillips EJ, Pilkinton MA, Craig-Owens L, Kola N, Plautz G, Reshef DS, Deutsch JS, Deering RP, Olenchock BA, Lichtman AH, Roden DM, Seidman CE, Koralnik IJ, Seidman JG, Hoffman RD, Taube JM, Diaz LA Jr, Anders RA, Sosman JA, Moslehi JJ (2016) Fulminant myocarditis with combination immune checkpoint blockade. N Engl J Med 375:1749–1755. 10.1056/NEJMoa1609214 27806233 10.1056/NEJMoa1609214 PMC5247797

[69] Johnson DB, Nebhan CA, Moslehi JJ, Balko JM (2022) Immune-checkpoint inhibitors: long-term implications of toxicity. Nat Rev Clin Oncol 19:254–267. 10.1038/s41571-022-00600-w 35082367 10.1038/s41571-022-00600-w PMC8790946

[70] Kahler KC, Hassel JC, Heinzerling L, Loquai C, Mossner R, Ugurel S, Zimmer L, Gutzmer R (2016) Management of side effects of immune checkpoint blockade by anti-CTLA-4 and anti-PD-1 antibodies in metastatic melanoma. J Dtsch Dermatol Ges 14:662–681. 10.1111/ddg.13047 27373241 10.1111/ddg.13047

[71] Karlstaedt A, Moslehi J, de Boer RA (2022) Cardio-onco-metabolism: metabolic remodelling in cardiovascular disease and cancer. Nat Rev Cardiol 19:414–425. 10.1038/s41569-022-00698-6 35440740 10.1038/s41569-022-00698-6 PMC10112835

[72] Karlstaedt A, Zhang X, Vitrac H, Harmancey R, Vasquez H, Wang JH, Goodell MA, Taegtmeyer H (2016) Oncometabolite d-2-hydroxyglutarate impairs alpha-ketoglutarate dehydrogenase and contractile function in rodent heart. Proc Natl Acad Sci U S A 113:10436–10441. 10.1073/pnas.1601650113 27582470 10.1073/pnas.1601650113 PMC5027422

[73] Karwi QG, Biswas D, Pulinilkunnil T, Lopaschuk GD (2020) Myocardial ketones metabolism in heart failure. J Card Fail 26:998–1005. 10.1016/j.cardfail.2020.04.005 32442517 10.1016/j.cardfail.2020.04.005

[74] Kattih B, Shirvani A, Klement P, Garrido AM, Gabdoulline R, Liebich A, Brandes M, Chaturvedi A, Seeger T, Thol F, Gohring G, Schlegelberger B, Geffers R, John D, Bavendiek U, Bauersachs J, Ganser A, Heineke J, Heuser M (2021) IDH1/2 mutations in acute myeloid leukemia patients and risk of coronary artery disease and cardiac dysfunction-a retrospective propensity score analysis. Leukemia 35:1301–1316. 10.1038/s41375-020-01043-x 32948843 10.1038/s41375-020-01043-x PMC8102189

[75] Kishton RJ, Sukumar M, Restifo NP (2017) Metabolic regulation of T cell longevity and function in tumor immunotherapy. Cell Metab 26:94–109. 10.1016/j.cmet.2017.06.016 28683298 10.1016/j.cmet.2017.06.016 PMC5543711

[76] Kolwicz SC Jr, Purohit S, Tian R (2013) Cardiac metabolism and its interactions with contraction, growth, and survival of cardiomyocytes. Circ Res 113:603–616. 10.1161/CIRCRESAHA.113.302095 23948585 10.1161/CIRCRESAHA.113.302095 PMC3845521

[77] Kranendijk M, Struys EA, Salomons GS, Van der Knaap MS, Jakobs C (2012) Progress in understanding 2-hydroxyglutaric acidurias. J Inherit Metab Dis 35:571–587. 10.1007/s10545-012-9462-5 22391998 10.1007/s10545-012-9462-5 PMC3388262

[78] Kuhnly NM, Coviello J (2022) Immune checkpoint inhibitor-related myocarditis: recognition, surveillance, and management. Clin J Oncol Nurs 26:54–60. 10.1188/22.CJON.54-60 35073300 10.1188/22.CJON.54-60

[79] Laenens D, Yu Y, Santens B, Jacobs J, Beuselinck B, Bechter O, Wauters E, Staessen J, Janssens S, Van Aelst L (2022) Incidence of cardiovascular events in patients treated with immune checkpoint inhibitors. J Clin Oncol 40:3430–3438. 10.1200/JCO.21.01808 35772044 10.1200/JCO.21.01808

[80] Lambert AW, Pattabiraman DR, Weinberg RA (2017) Emerging biological principles of metastasis. Cell 168:670–691. 10.1016/j.cell.2016.11.037 28187288 10.1016/j.cell.2016.11.037 PMC5308465

[81] Le MT, Frye RF, Rivard CJ, Cheng J, McFann KK, Segal MS, Johnson RJ, Johnson JA (2012) Effects of high-fructose corn syrup and sucrose on the pharmacokinetics of fructose and acute metabolic and hemodynamic responses in healthy subjects. Metabolism 61:641–651. 10.1016/j.metabol.2011.09.013 22152650 10.1016/j.metabol.2011.09.013 PMC3306467

[82] Lehmann LH, Heckmann MB, Bailly G, Finke D, Procureur A, Power JR, Stein F, Bretagne M, Ederhy S, Fenioux C, Hamwy O, Funck-Brentano E, Romano E, Pieroni L, Munster JP, Allenbach Y, Anquetil C, Leonard-Louis S, Palaskas NL, Hayek SS, Katus HA, Giannitsis E, Frey N, Kaya Z, Moslehi J, Prifti E, Salem JE (2023) Cardiomuscular biomarkers in the diagnosis and prognostication of immune checkpoint inhibitor myocarditis. Circulation 148:473–486. 10.1161/CIRCULATIONAHA.123.062405 37317858 10.1161/CIRCULATIONAHA.123.062405 PMC10527069

[83] Li L, Niemann B, Knapp F, Werner S, Muhlfeld C, Schneider JP, Jurida LM, Molenda N, Schmitz ML, Yin X, Mayr M, Schulz R, Kracht M, Rohrbach S (2024) Comparison of the stage-dependent mitochondrial changes in response to pressure overload between the diseased right and left ventricle in the rat. Basic Res Cardiol 119:587–611. 10.1007/s00395-024-01051-3 38758338 10.1007/s00395-024-01051-3

[84] Li Y, Wu Y, Hu Y (2021) Metabolites in the tumor microenvironment reprogram functions of immune effector cells through epigenetic modifications. Front Immunol. 10.3389/fimmu.2021.641883 33927716 10.3389/fimmu.2021.641883 PMC8078775

[85] Liao P, Wang W, Wang W, Kryczek I, Li X, Bian Y, Sell A, Wei S, Grove S, Johnson JK, Kennedy PD, Gijon M, Shah YM, Zou W (2022) CD8(+) T cells and fatty acids orchestrate tumor ferroptosis and immunity via ACSL4. Cancer Cell. 10.1016/j.ccell.2022.02.003 35216678 10.1016/j.ccell.2022.02.003 PMC9007863

[86] Liu G, Chen T, Zhang X, Hu B, Shi H (2024) Immune checkpoint inhibitor-associated cardiovascular toxicities: a review. Heliyon. 10.1016/j.heliyon.2024.e25747 38434280 10.1016/j.heliyon.2024.e25747 PMC10907684

[87] Lopaschuk GD, Karwi QG, Tian R, Wende AR, Abel ED (2021) Cardiac energy metabolism in heart failure. Circ Res 128:1487–1513. 10.1161/CIRCRESAHA.121.318241 33983836 10.1161/CIRCRESAHA.121.318241 PMC8136750

[88] Lopaschuk GD, Ussher JR, Folmes CD, Jaswal JS, Stanley WC (2010) Myocardial fatty acid metabolism in health and disease. Physiol Rev 90:207–258. 10.1152/physrev.00015.2009 20086077 10.1152/physrev.00015.2009

[89] Losman JA, Looper RE, Koivunen P, Lee S, Schneider RK, McMahon C, Cowley GS, Root DE, Ebert BL, Kaelin WG Jr (2013) (R)-2-hydroxyglutarate is sufficient to promote leukemogenesis and its effects are reversible. Science 339:1621–1625. 10.1126/science.1231677 23393090 10.1126/science.1231677 PMC3836459

[90] Lucas JA, Menke J, Rabacal WA, Schoen FJ, Sharpe AH, Kelley VR (2008) Programmed death ligand 1 regulates a critical checkpoint for autoimmune myocarditis and pneumonitis in MRL mice. J Immunol 181:2513–2521. 10.4049/jimmunol.181.4.2513 18684942 10.4049/jimmunol.181.4.2513 PMC2587295

[91] Luengo A, Gui DY, Vander Heiden MG (2017) Targeting metabolism for cancer therapy. Cell Chem Biol 24:1161–1180. 10.1016/j.chembiol.2017.08.028 28938091 10.1016/j.chembiol.2017.08.028 PMC5744685

[92] Lyon AR, Lopez-Fernandez T, Couch LS, Asteggiano R, Aznar MC, Bergler-Klein J, Boriani G, Cardinale D, Cordoba R, Cosyns B, Cutter DJ, de Azambuja E, de Boer RA, Dent SF, Farmakis D, Gevaert SA, Gorog DA, Herrmann J, Lenihan D, Moslehi J, Moura B, Salinger SS, Stephens R, Suter TM, Szmit S, Tamargo J, Thavendiranathan P, Tocchetti CG, van der Meer P, van der Pal HJH, Group ESCSD (2022) 2022 ESC Guidelines on cardio-oncology developed in collaboration with the European Hematology Association (EHA), the European Society for Therapeutic Radiology and Oncology (ESTRO) and the International Cardio-Oncology Society (IC-OS). Eur Heart J Cardiovasc Imaging 23:e333–e465. 10.1093/ehjci/jeac106 36017575 10.1093/ehjci/jeac106

[93] Lyon AR, Yousaf N, Battisti NML, Moslehi J, Larkin J (2018) Immune checkpoint inhibitors and cardiovascular toxicity. Lancet Oncol 19:e447–e458. 10.1016/S1470-2045(18)30457-1 30191849 10.1016/S1470-2045(18)30457-1

[94] Ma X, Xiao L, Liu L, Ye L, Su P, Bi E, Wang Q, Yang M, Qian J, Yi Q (2021) CD36-mediated ferroptosis dampens intratumoral CD8(+) T cell effector function and impairs their antitumor ability. Cell Metab. 10.1016/j.cmet.2021.02.015 33691090 10.1016/j.cmet.2021.02.015 PMC8102368

[95] Madden MZ, Rathmell JC (2021) The complex integration of T-cell metabolism and immunotherapy. Cancer Discov 11:1636–1643. 10.1158/2159-8290.CD-20-0569 33795235 10.1158/2159-8290.CD-20-0569 PMC8295173

[96] Mahmood SS, Fradley MG, Cohen JV, Nohria A, Reynolds KL, Heinzerling LM, Sullivan RJ, Damrongwatanasuk R, Chen CL, Gupta D, Kirchberger MC, Awadalla M, Hassan MZO, Moslehi JJ, Shah SP, Ganatra S, Thavendiranathan P, Lawrence DP, Groarke JD, Neilan TG (2018) Myocarditis in patients treated with immune checkpoint inhibitors. J Am Coll Cardiol 71:1755–1764. 10.1016/j.jacc.2018.02.037 29567210 10.1016/j.jacc.2018.02.037 PMC6196725

[97] Malekan M, Ebrahimzadeh MA, Sheida F (2021) The role of Hypoxia-Inducible Factor-1alpha and its signaling in melanoma. Biomed Pharmacother 141:111873. 10.1016/j.biopha.2021.111873 34225012 10.1016/j.biopha.2021.111873

[98] Martinez-Reyes I, Chandel NS (2021) Cancer metabolism: looking forward. Nat Rev Cancer 21:669–680. 10.1038/s41568-021-00378-6 34272515 10.1038/s41568-021-00378-6

[99] Martins F, Sofiya L, Sykiotis GP, Lamine F, Maillard M, Fraga M, Shabafrouz K, Ribi C, Cairoli A, Guex-Crosier Y, Kuntzer T, Michielin O, Peters S, Coukos G, Spertini F, Thompson JA, Obeid M (2019) Adverse effects of immune-checkpoint inhibitors: epidemiology, management and surveillance. Nat Rev Clin Oncol 16:563–580. 10.1038/s41571-019-0218-0 31092901 10.1038/s41571-019-0218-0

[100] Mayers JR, Torrence ME, Danai LV, Papagiannakopoulos T, Davidson SM, Bauer MR, Lau AN, Ji BW, Dixit PD, Hosios AM, Muir A, Chin CR, Freinkman E, Jacks T, Wolpin BM, Vitkup D, Vander Heiden MG (2016) Tissue of origin dictates branched-chain amino acid metabolism in mutant Kras-driven cancers. Science 353:1161–1165. 10.1126/science.aaf5171 27609895 10.1126/science.aaf5171 PMC5245791

[101] McNulty PH, Jacob R, Deckelbaum LI, Young LH (2000) Effect of hyperinsulinemia on myocardial amino acid uptake in patients with coronary artery disease. Metabolism 49:1365–1369. 10.1053/meta.2000.9510 11079831 10.1053/meta.2000.9510

[102] Michel L, Ferdinandy P, Rassaf T (2024) Cellular alterations in immune checkpoint inhibitor therapy-related cardiac dysfunction. Curr Heart Fail Rep 21:214–223. 10.1007/s11897-024-00652-2 38430308 10.1007/s11897-024-00652-2 PMC11090976

[103] Michel L, Helfrich I, Hendgen-Cotta UB, Mincu RI, Korste S, Mrotzek SM, Spomer A, Odersky A, Rischpler C, Herrmann K, Umutlu L, Coman C, Ahrends R, Sickmann A, Loffek S, Livingstone E, Ugurel S, Zimmer L, Gunzer M, Schadendorf D, Totzeck M, Rassaf T (2022) Targeting early stages of cardiotoxicity from anti-PD1 immune checkpoint inhibitor therapy. Eur Heart J 43:316–329. 10.1093/eurheartj/ehab430 34389849 10.1093/eurheartj/ehab430

[104] Miranda-Silva D, Lima T, Rodrigues P, Leite-Moreira A, Falcao-Pires I (2021) Mechanisms underlying the pathophysiology of heart failure with preserved ejection fraction: the tip of the iceberg. Heart Fail Rev 26:453–478. 10.1007/s10741-020-10042-0 33411091 10.1007/s10741-020-10042-0

[105] Moore SF, van den Bosch MT, Hunter RW, Sakamoto K, Poole AW, Hers I (2013) Dual regulation of glycogen synthase kinase 3 (GSK3)alpha/beta by protein kinase C (PKC)alpha and Akt promotes thrombin-mediated integrin alphaIIbbeta3 activation and granule secretion in platelets. J Biol Chem 288:3918–3928. 10.1074/jbc.M112.429936 23239877 10.1074/jbc.M112.429936 PMC3567645

[106] Moslehi JJ, Salem JE, Sosman JA, Lebrun-Vignes B, Johnson DB (2018) Increased reporting of fatal immune checkpoint inhibitor-associated myocarditis. Lancet 391:933. 10.1016/S0140-6736(18)30533-6 29536852 10.1016/S0140-6736(18)30533-6 PMC6668330

[107] Mueckler M, Thorens B (2013) The SLC2 (GLUT) family of membrane transporters. Mol Aspects Med 34:121–138. 10.1016/j.mam.2012.07.001 23506862 10.1016/j.mam.2012.07.001 PMC4104978

[108] Murashige D, Jang C, Neinast M, Edwards JJ, Cowan A, Hyman MC, Rabinowitz JD, Frankel DS, Arany Z (2020) Comprehensive quantification of fuel use by the failing and nonfailing human heart. Science 370:364–368. 10.1126/science.abc8861 33060364 10.1126/science.abc8861 PMC7871704

[109] Murthy MS, Pande SV (1987) Malonyl-CoA binding site and the overt carnitine palmitoyltransferase activity reside on the opposite sides of the outer mitochondrial membrane. Proc Natl Acad Sci U S A 84:378–382. 10.1073/pnas.84.2.378 3540964 10.1073/pnas.84.2.378 PMC304210

[110] Nakaya M, Xiao Y, Zhou X, Chang JH, Chang M, Cheng X, Blonska M, Lin X, Sun SC (2014) Inflammatory T cell responses rely on amino acid transporter ASCT2 facilitation of glutamine uptake and mTORC1 kinase activation. Immunity 40:692–705. 10.1016/j.immuni.2014.04.007 24792914 10.1016/j.immuni.2014.04.007 PMC4074507

[111] Nascentes Melo LM, Lesner NP, Sabatier M, Ubellacker JM, Tasdogan A (2022) Emerging metabolomic tools to study cancer metastasis. Trends Cancer 8:988–1001. 10.1016/j.trecan.2022.07.003 35909026 10.1016/j.trecan.2022.07.003

[112] Neely JR, Morgan HE (1974) Relationship between carbohydrate and lipid metabolism and the energy balance of heart muscle. Annu Rev Physiol 36:413–459. 10.1146/annurev.ph.36.030174.002213 19400669 10.1146/annurev.ph.36.030174.002213

[113] Neilan TG, Rothenberg ML, Amiri-Kordestani L, Sullivan RJ, Steingart RM, Gregory W, Hariharan S, Hammad TA, Lindenfeld J, Murphy MJ, Moslehi JJ, Checkpoint Inhibitor Safety Working G (2018) Myocarditis associated with immune checkpoint inhibitors: an expert consensus on data gaps and a call to action. Oncologist 23:874–878. 10.1634/theoncologist.2018-0157 29802220 10.1634/theoncologist.2018-0157 PMC6156187

[114] Neinast MD, Jang C, Hui S, Murashige DS, Chu Q, Morscher RJ, Li X, Zhan L, White E, Anthony TG, Rabinowitz JD, Arany Z (2019) Quantitative analysis of the whole-body metabolic fate of branched-chain amino acids. Cell Metab. 10.1016/j.cmet.2018.10.013 30449684 10.1016/j.cmet.2018.10.013 PMC6365191

[115] Norwood TG, Westbrook BC, Johnson DB, Litovsky SH, Terry NL, McKee SB, Gertler AS, Moslehi JJ, Conry RM (2017) Smoldering myocarditis following immune checkpoint blockade. J Immunother Cancer 5:91. 10.1186/s40425-017-0296-4 29157297 10.1186/s40425-017-0296-4 PMC5697345

[116] Ogando J, Saez ME, Santos J, Nuevo-Tapioles C, Gut M, Esteve-Codina A, Heath S, Gonzalez-Perez A, Cuezva JM, Lacalle RA, Manes S (2019) PD-1 signaling affects cristae morphology and leads to mitochondrial dysfunction in human CD8(+) T lymphocytes. J Immunother Cancer 7:151. 10.1186/s40425-019-0628-7 31196176 10.1186/s40425-019-0628-7 PMC6567413

[117] Okazaki T, Tanaka Y, Nishio R, Mitsuiye T, Mizoguchi A, Wang J, Ishida M, Hiai H, Matsumori A, Minato N, Honjo T (2003) Autoantibodies against cardiac troponin I are responsible for dilated cardiomyopathy in PD-1-deficient mice. Nat Med 9:1477–1483. 10.1038/nm955 14595408 10.1038/nm955

[118] Osuna-Prieto FJ, Martinez-Tellez B, Ortiz-Alvarez L, Di X, Jurado-Fasoli L, Xu H, Ceperuelo-Mallafre V, Nunez-Roa C, Kohler I, Segura-Carretero A, Garcia-Lario JV, Gil A, Aguilera CM, Llamas-Elvira JM, Rensen PCN, Vendrell J, Ruiz JR, Fernandez-Veledo S (2021) Elevated plasma succinate levels are linked to higher cardiovascular disease risk factors in young adults. Cardiovasc Diabetol 20:151. 10.1186/s12933-021-01333-3 34315463 10.1186/s12933-021-01333-3 PMC8314524

[119] Park J, Hsueh PC, Li Z, Ho PC (2023) Microenvironment-driven metabolic adaptations guiding CD8(+) T cell anti-tumor immunity. Immunity 56:32–42. 10.1016/j.immuni.2022.12.008 36630916 10.1016/j.immuni.2022.12.008

[120] Park J, Wang L, Ho PC (2022) Metabolic guidance and stress in tumors modulate antigen-presenting cells. Oncogenesis 11:62. 10.1038/s41389-022-00438-y 36244976 10.1038/s41389-022-00438-y PMC9573874

[121] Parker SJ, Metallo CM (2015) Metabolic consequences of oncogenic IDH mutations. Pharmacol Ther 152:54–62. 10.1016/j.pharmthera.2015.05.003 25956465 10.1016/j.pharmthera.2015.05.003 PMC4489982

[122] Parry RV, Chemnitz JM, Frauwirth KA, Lanfranco AR, Braunstein I, Kobayashi SV, Linsley PS, Thompson CB, Riley JL (2005) CTLA-4 and PD-1 receptors inhibit T-cell activation by distinct mechanisms. Mol Cell Biol 25:9543–9553. 10.1128/MCB.25.21.9543-9553.2005 16227604 10.1128/MCB.25.21.9543-9553.2005 PMC1265804

[123] Patsoukis N, Bardhan K, Chatterjee P, Sari D, Liu B, Bell LN, Karoly ED, Freeman GJ, Petkova V, Seth P, Li L, Boussiotis VA (2015) PD-1 alters T-cell metabolic reprogramming by inhibiting glycolysis and promoting lipolysis and fatty acid oxidation. Nat Commun 6:6692. 10.1038/ncomms7692 25809635 10.1038/ncomms7692 PMC4389235

[124] Pavlova NN, Thompson CB (2016) The emerging hallmarks of cancer metabolism. Cell Metab 23:27–47. 10.1016/j.cmet.2015.12.006 26771115 10.1016/j.cmet.2015.12.006 PMC4715268

[125] Pavlova NN, Zhu J, Thompson CB (2022) The hallmarks of cancer metabolism: still emerging. Cell Metab 34:355–377. 10.1016/j.cmet.2022.01.007 35123658 10.1016/j.cmet.2022.01.007 PMC8891094

[126] Peranzoni E, Lemoine J, Vimeux L, Feuillet V, Barrin S, Kantari-Mimoun C, Bercovici N, Guerin M, Biton J, Ouakrim H, Regnier F, Lupo A, Alifano M, Damotte D, Donnadieu E (2018) Macrophages impede CD8 T cells from reaching tumor cells and limit the efficacy of anti-PD-1 treatment. Proc Natl Acad Sci U S A 115:E4041–E4050. 10.1073/pnas.1720948115 29632196 10.1073/pnas.1720948115 PMC5924916

[127] Poznanski SM, Singh K, Ritchie TM, Aguiar JA, Fan IY, Portillo AL, Rojas EA, Vahedi F, El-Sayes A, Xing S, Butcher M, Lu Y, Doxey AC, Schertzer JD, Hirte HW, Ashkar AA (2021) Metabolic flexibility determines human NK cell functional fate in the tumor microenvironment. Cell Metab. 10.1016/j.cmet.2021.03.023 33852875 10.1016/j.cmet.2021.03.023

[128] Prag HA, Gruszczyk AV, Huang MM, Beach TE, Young T, Tronci L, Nikitopoulou E, Mulvey JF, Ascione R, Hadjihambi A, Shattock MJ, Pellerin L, Saeb-Parsy K, Frezza C, James AM, Krieg T, Murphy MP, Aksentijevic D (2021) Mechanism of succinate efflux upon reperfusion of the ischaemic heart. Cardiovasc Res 117:1188–1201. 10.1093/cvr/cvaa148 32766828 10.1093/cvr/cvaa148 PMC7983001

[129] Qorraj M, Bruns H, Bottcher M, Weigand L, Saul D, Mackensen A, Jitschin R, Mougiakakos D (2017) The PD-1/PD-L1 axis contributes to immune metabolic dysfunctions of monocytes in chronic lymphocytic leukemia. Leukemia 31:470–478. 10.1038/leu.2016.214 27479178 10.1038/leu.2016.214

[130] Quagliariello V, Passariello M, Rea D, Barbieri A, Iovine M, Bonelli A, Caronna A, Botti G, De Lorenzo C, Maurea N (2020) Evidences of CTLA-4 and PD-1 blocking agents-induced cardiotoxicity in cellular and preclinical models. J Pers Med. 10.3390/jpm10040179 33086484 10.3390/jpm10040179 PMC7711520

[131] Rabinowitz JD, Enerback S (2020) Lactate: the ugly duckling of energy metabolism. Nat Metab 2:566–571. 10.1038/s42255-020-0243-4 32694798 10.1038/s42255-020-0243-4 PMC7983055

[132] Reichmann H, Angelini C (1994) Single muscle fibre analyses in 2 brothers with succinate dehydrogenase deficiency. Eur Neurol 34:95–98. 10.1159/000117016 8174601 10.1159/000117016

[133] Rice CM, Davies LC, Subleski JJ, Maio N, Gonzalez-Cotto M, Andrews C, Patel NL, Palmieri EM, Weiss JM, Lee JM, Annunziata CM, Rouault TA, Durum SK, McVicar DW (2018) Tumour-elicited neutrophils engage mitochondrial metabolism to circumvent nutrient limitations and maintain immune suppression. Nat Commun 9:5099. 10.1038/s41467-018-07505-2 30504842 10.1038/s41467-018-07505-2 PMC6269473

[134] Ritterhoff J, Young S, Villet O, Shao D, Neto FC, Bettcher LF, Hsu YA, Kolwicz SC Jr, Raftery D, Tian R (2020) Metabolic remodeling promotes cardiac hypertrophy by directing glucose to aspartate biosynthesis. Circ Res 126:182–196. 10.1161/CIRCRESAHA.119.315483 31709908 10.1161/CIRCRESAHA.119.315483 PMC8448129

[135] Roth ME, Muluneh B, Jensen BC, Madamanchi C, Lee CB (2016) Left ventricular dysfunction after treatment with ipilimumab for metastatic melanoma. Am J Ther 23:e1925–e1928. 10.1097/MJT.0000000000000430 26885708 10.1097/MJT.0000000000000430

[136] Rustin P, Lebidois J, Chretien D, Bourgeron T, Piechaud JF, Rotig A, Sidi D, Munnich A (1993) The investigation of respiratory chain disorders in heart using endomyocardial biopsies. J Inherit Metab Dis 16:541–544. 10.1007/BF00711676 7609447 10.1007/BF00711676

[137] Salem JE, Bretagne M, Abbar B, Leonard-Louis S, Ederhy S, Redheuil A, Boussouar S, Nguyen LS, Procureur A, Stein F, Fenioux C, Devos P, Gougis P, Dres M, Demoule A, Psimaras D, Lenglet T, Maisonobe T, De Chambrun MP, Hekimian G, Straus C, Gonzalez-Bermejo J, Klatzmann D, Rigolet A, Guillaume-Jugnot P, Champtiaux N, Benveniste O, Weiss N, Saheb S, Rouvier P, Plu I, Gandjbakhch E, Kerneis M, Hammoudi N, Zahr N, Llontop C, Morelot-Panzini C, Lehmann L, Qin J, Moslehi JJ, Rosenzwajg M, Similowski T, Allenbach Y (2023) Abatacept/ruxolitinib and screening for concomitant respiratory muscle failure to mitigate fatality of immune-checkpoint inhibitor myocarditis. Cancer Discov 13:1100–1115. 10.1158/2159-8290.CD-22-1180 36815259 10.1158/2159-8290.CD-22-1180

[138] Salem JE, Manouchehri A, Moey M, Lebrun-Vignes B, Bastarache L, Pariente A, Gobert A, Spano JP, Balko JM, Bonaca MP, Roden DM, Johnson DB, Moslehi JJ (2018) Cardiovascular toxicities associated with immune checkpoint inhibitors: an observational, retrospective, pharmacovigilance study. Lancet Oncol 19:1579–1589. 10.1016/S1470-2045(18)30608-9 30442497 10.1016/S1470-2045(18)30608-9 PMC6287923

[139] Salzberger W, Martrus G, Bachmann K, Goebels H, Hess L, Koch M, Langeneckert A, Lunemann S, Oldhafer KJ, Pfeifer C, Poch T, Richert L, Schramm C, Wahib R, Bunders MJ, Altfeld M (2018) Tissue-resident NK cells differ in their expression profile of the nutrient transporters Glut1, CD98 and CD71. PLoS ONE. 10.1371/journal.pone.0201170 30028872 10.1371/journal.pone.0201170 PMC6054388

[140] Scharping NE, Menk AV, Moreci RS, Whetstone RD, Dadey RE, Watkins SC, Ferris RL, Delgoffe GM (2016) The tumor microenvironment represses T cell mitochondrial biogenesis to drive intratumoral T cell metabolic insufficiency and dysfunction. Immunity 45:374–388. 10.1016/j.immuni.2016.07.009 27496732 10.1016/j.immuni.2016.07.009 PMC5207350

[141] Scharping NE, Rivadeneira DB, Menk AV, Vignali PDA, Ford BR, Rittenhouse NL, Peralta R, Wang Y, Wang Y, DePeaux K, Poholek AC, Delgoffe GM (2021) Mitochondrial stress induced by continuous stimulation under hypoxia rapidly drives T cell exhaustion. Nat Immunol 22:205–215. 10.1038/s41590-020-00834-9 33398183 10.1038/s41590-020-00834-9 PMC7971090

[142] Schumacker PT (2006) Reactive oxygen species in cancer cells: live by the sword, die by the sword. Cancer Cell 10:175–176. 10.1016/j.ccr.2006.08.015 16959608 10.1016/j.ccr.2006.08.015

[143] Sedlackova L, Korolchuk VI (2019) Mitochondrial quality control as a key determinant of cell survival. Biochim Biophys Acta Mol Cell Res 1866:575–587. 10.1016/j.bbamcr.2018.12.012 30594496 10.1016/j.bbamcr.2018.12.012

[144] Shapira-Frommer R, Niu J, Perets R, Peters S, Shouse G, Lugowska I, Garassino MC, Sands J, Keenan T, Zhao B, Healy J (2024) Ahn MJ (2024) The KEYVIBE program: vibostolimab and pembrolizumab for the treatment of advanced malignancies. Future Oncol. 10.1080/14796694.2024.2343272 39230120 10.1080/14796694.2024.2343272 PMC11497960

[145] Skinner R, Trujillo A, Ma X, Beierle EA (2009) Ketone bodies inhibit the viability of human neuroblastoma cells. J Pediatr Surg 44:212–216. 10.1016/j.jpedsurg.2008.10.042 19159745 10.1016/j.jpedsurg.2008.10.042

[146] Spain L, Diem S, Larkin J (2016) Management of toxicities of immune checkpoint inhibitors. Cancer Treat Rev 44:51–60. 10.1016/j.ctrv.2016.02.001 26874776 10.1016/j.ctrv.2016.02.001

[147] Suero-Abreu GA, Zanni MV, Neilan TG (2022) Atherosclerosis With immune checkpoint inhibitor therapy: evidence, diagnosis, and management: JACC: CardioOncology state-of-the-art review. JACC CardioOncol 4:598–615. 10.1016/j.jaccao.2022.11.011 36636438 10.1016/j.jaccao.2022.11.011 PMC9830225

[148] Sugiura A, Rathmell JC (2018) Metabolic barriers to T cell function in tumors. J Immunol 200:400–407. 10.4049/jimmunol.1701041 29311381 10.4049/jimmunol.1701041 PMC5777533

[149] Swinnen JV, Brusselmans K, Verhoeven G (2006) Increased lipogenesis in cancer cells: new players, novel targets. Curr Opin Clin Nutr Metab Care 9:358–365. 10.1097/01.mco.0000232894.28674.30 16778563 10.1097/01.mco.0000232894.28674.30

[150] Tang T, Huang X, Zhang G, Hong Z, Bai X, Liang T (2021) Advantages of targeting the tumor immune microenvironment over blocking immune checkpoint in cancer immunotherapy. Signal Transduct Target Ther 6:72. 10.1038/s41392-020-00449-4 33608497 10.1038/s41392-020-00449-4 PMC7896069

[151] Tarrio ML, Grabie N, Bu DX, Sharpe AH, Lichtman AH (2012) PD-1 protects against inflammation and myocyte damage in T cell-mediated myocarditis. J Immunol 188:4876–4884. 10.4049/jimmunol.1200389 22491251 10.4049/jimmunol.1200389 PMC3345066

[152] Tawbi HA, Schadendorf D, Lipson EJ, Ascierto PA, Matamala L, Castillo Gutierrez E, Rutkowski P, Gogas HJ, Lao CD, De Menezes JJ, Dalle S, Arance A, Grob JJ, Srivastava S, Abaskharoun M, Hamilton M, Keidel S, Simonsen KL, Sobiesk AM, Li B, Hodi FS, Long GV, Investigators R (2022) Relatlimab and nivolumab versus nivolumab in untreated advanced melanoma. N Engl J Med 386:24–34. 10.1056/NEJMoa2109970 34986285 10.1056/NEJMoa2109970 PMC9844513

[153] Taylor SR, Ramsamooj S, Liang RJ, Katti A, Pozovskiy R, Vasan N, Hwang SK, Nahiyaan N, Francoeur NJ, Schatoff EM, Johnson JL, Shah MA, Dannenberg AJ, Sebra RP, Dow LE, Cantley LC, Rhee KY, Goncalves MD (2021) Dietary fructose improves intestinal cell survival and nutrient absorption. Nature 597:263–267. 10.1038/s41586-021-03827-2 34408323 10.1038/s41586-021-03827-2 PMC8686685

[154] Thorens B, Mueckler M (2010) Glucose transporters in the 21st Century. Am J Physiol Endocrinol Metab 298:E141-145. 10.1152/ajpendo.00712.2009 20009031 10.1152/ajpendo.00712.2009 PMC2822486

[155] Tkachev V, Goodell S, Opipari AW, Hao LY, Franchi L, Glick GD, Ferrara JL, Byersdorfer CA (2015) Programmed death-1 controls T cell survival by regulating oxidative metabolism. J Immunol 194:5789–5800. 10.4049/jimmunol.1402180 25972478 10.4049/jimmunol.1402180 PMC4562423

[156] Tomlinson IP, Alam NA, Rowan AJ, Barclay E, Jaeger EE, Kelsell D, Leigh I, Gorman P, Lamlum H, Rahman S, Roylance RR, Olpin S, Bevan S, Barker K, Hearle N, Houlston RS, Kiuru M, Lehtonen R, Karhu A, Vilkki S, Laiho P, Eklund C, Vierimaa O, Aittomaki K, Hietala M, Sistonen P, Paetau A, Salovaara R, Herva R, Launonen V, Aaltonen LA, Multiple Leiomyoma C (2002) Germline mutations in FH predispose to dominantly inherited uterine fibroids, skin leiomyomata and papillary renal cell cancer. Nat Genet 30:406–410. 10.1038/ng849 11865300 10.1038/ng849

[157] Tschopp J, Schroder K (2010) NLRP3 inflammasome activation: the convergence of multiple signalling pathways on ROS production? Nat Rev Immunol 10:210–215. 10.1038/nri2725 20168318 10.1038/nri2725

[158] Umbarawan Y, Syamsunarno M, Koitabashi N, Yamaguchi A, Hanaoka H, Hishiki T, Nagahata-Naito Y, Obinata H, Sano M, Sunaga H, Matsui H, Tsushima Y, Suematsu M, Kurabayashi M, Iso T (2018) Glucose is preferentially utilized for biomass synthesis in pressure-overloaded hearts: evidence from fatty acid-binding protein-4 and -5 knockout mice. Cardiovasc Res 114:1132–1144. 10.1093/cvr/cvy063 29554241 10.1093/cvr/cvy063 PMC6014234

[159] Vander Heiden MG, DeBerardinis RJ (2017) Understanding the intersections between metabolism and cancer biology. Cell 168:657–669. 10.1016/j.cell.2016.12.039 28187287 10.1016/j.cell.2016.12.039 PMC5329766

[160] Veech RL, Chance B, Kashiwaya Y, Lardy HA, Cahill GF Jr (2001) Ketone bodies, potential therapeutic uses. IUBMB Life 51:241–247. 10.1080/152165401753311780 11569918 10.1080/152165401753311780

[161] Vernieri C, Ligorio F, Dieci MV, Lambertini M, De Angelis C, Iorfida M, Botticelli A, Strina C, Vingiani A, Provenzano L, Bianchi GV, Folli S, Generali D, Zambelli A, de Placido S, Del Mastro L, Guarneri V, Capri G, Pruneri G, De Braud FGM (2023) 352TiP Neoadjuvant chemo-immunotherapy plus/minus fasting-like approach in stage II-III triple-negative breast cancer patients: the phase II randomized BREAKFAST-2 trial. Ann Oncol 34:S322–S323. 10.1016/j.annonc.2023.09.2829

[162] Waliany S, Lee D, Witteles RM, Neal JW, Nguyen P, Davis MM, Salem JE, Wu SM, Moslehi JJ, Zhu H (2021) Immune checkpoint inhibitor cardiotoxicity: understanding basic mechanisms and clinical characteristics and finding a cure. Annu Rev Pharmacol Toxicol 61:113–134. 10.1146/annurev-pharmtox-010919-023451 32776859 10.1146/annurev-pharmtox-010919-023451

[163] Wang C, Qin L, Manes TD, Kirkiles-Smith NC, Tellides G, Pober JS (2014) Rapamycin antagonizes TNF induction of VCAM-1 on endothelial cells by inhibiting mTORC2. J Exp Med 211:395–404. 10.1084/jem.20131125 24516119 10.1084/jem.20131125 PMC3949571

[164] Wang R, Dillon CP, Shi LZ, Milasta S, Carter R, Finkelstein D, McCormick LL, Fitzgerald P, Chi H, Munger J, Green DR (2011) The transcription factor Myc controls metabolic reprogramming upon T lymphocyte activation. Immunity 35:871–882. 10.1016/j.immuni.2011.09.021 22195744 10.1016/j.immuni.2011.09.021 PMC3248798

[165] Warburg O (1956) On respiratory impairment in cancer cells. Science 124:269–270 13351639

[166] Warburg O (1956) On the origin of cancer cells. Science 123:309–314. 10.1126/science.123.3191.309 13298683 10.1126/science.123.3191.309

[167] Warburg O, Wind F, Negelein E (1927) The metabolism of tumors in the body. J Gen Physiol 8:519–530. 10.1085/jgp.8.6.519 19872213 10.1085/jgp.8.6.519 PMC2140820

[168] Waterhouse P, Penninger JM, Timms E, Wakeham A, Shahinian A, Lee KP, Thompson CB, Griesser H, Mak TW (1995) Lymphoproliferative disorders with early lethality in mice deficient in Ctla-4. Science 270:985–988. 10.1126/science.270.5238.985 7481803 10.1126/science.270.5238.985

[169] Wei SC, Meijers WC, Axelrod ML, Anang NAS, Screever EM, Wescott EC, Johnson DB, Whitley E, Lehmann L, Courand PY, Mancuso JJ, Himmel LE, Lebrun-Vignes B, Wleklinski MJ, Knollmann BC, Srinivasan J, Li Y, Atolagbe OT, Rao X, Zhao Y, Wang J, Ehrlich LIR, Sharma P, Salem JE, Balko JM, Moslehi JJ, Allison JP (2021) A genetic mouse model recapitulates immune checkpoint inhibitor-associated myocarditis and supports a mechanism-based therapeutic intervention. Cancer Discov 11:614–625. 10.1158/2159-8290.CD-20-0856 33257470 10.1158/2159-8290.CD-20-0856 PMC8041233

[170] Weinberg F, Ramnath N, Nagrath D (2019) Reactive oxygen species in the tumor microenvironment: an overview. Cancers (Basel). 10.3390/cancers11081191 31426364 10.3390/cancers11081191 PMC6721577

[171] Weng Y, Fan X, Bai Y, Wang S, Huang H, Yang H, Zhu J, Zhang F (2018) SLC2A5 promotes lung adenocarcinoma cell growth and metastasis by enhancing fructose utilization. Cell Death Discov 4:38. 10.1038/s41420-018-0038-5 29531835 10.1038/s41420-018-0038-5 PMC5841403

[172] Wentz AE, d’Avignon DA, Weber ML, Cotter DG, Doherty JM, Kerns R, Nagarajan R, Reddy N, Sambandam N, Crawford PA (2010) Adaptation of myocardial substrate metabolism to a ketogenic nutrient environment. J Biol Chem 285:24447–24456. 10.1074/jbc.M110.100651 20529848 10.1074/jbc.M110.100651 PMC2915681

[173] Wolchok JD, Chiarion-Sileni V, Gonzalez R, Rutkowski P, Grob JJ, Cowey CL, Lao CD, Wagstaff J, Schadendorf D, Ferrucci PF, Smylie M, Dummer R, Hill A, Hogg D, Haanen J, Carlino MS, Bechter O, Maio M, Marquez-Rodas I, Guidoboni M, McArthur G, Lebbe C, Ascierto PA, Long GV, Cebon J, Sosman J, Postow MA, Callahan MK, Walker D, Rollin L, Bhore R, Hodi FS, Larkin J (2017) Overall survival with combined nivolumab and ipilimumab in advanced melanoma. N Engl J Med 377:1345–1356. 10.1056/NEJMoa1709684 28889792 10.1056/NEJMoa1709684 PMC5706778

[174] Wu G, Fang YZ, Yang S, Lupton JR, Turner ND (2004) Glutathione metabolism and its implications for health. J Nutr 134:489–492. 10.1093/jn/134.3.489 14988435 10.1093/jn/134.3.489

[175] Wu GY, Thompson JR (1988) The effect of ketone bodies on alanine and glutamine metabolism in isolated skeletal muscle from the fasted chick. Biochem J 255:139–144. 10.1042/bj2550139 2904261 10.1042/bj2550139 PMC1135201

[176] Yan J, Yan JY, Wang YX, Ling YN, Song XD, Wang SY, Liu HQ, Liu QC, Zhang Y, Yang PZ, Wang XB, Chen AH (2019) Spermidine-enhanced autophagic flux improves cardiac dysfunction following myocardial infarction by targeting the AMPK/mTOR signalling pathway. Br J Pharmacol 176:3126–3142. 10.1111/bph.14706 31077347 10.1111/bph.14706 PMC6692641

[177] Yang W, Bai Y, Xiong Y, Zhang J, Chen S, Zheng X, Meng X, Li L, Wang J, Xu C, Yan C, Wang L, Chang CC, Chang TY, Zhang T, Zhou P, Song BL, Liu W, Sun SC, Liu X, Li BL, Xu C (2016) Potentiating the antitumour response of CD8(+) T cells by modulating cholesterol metabolism. Nature 531:651–655. 10.1038/nature17412 26982734 10.1038/nature17412 PMC4851431

[178] Yatsunenko T, Rey FE, Manary MJ, Trehan I, Dominguez-Bello MG, Contreras M, Magris M, Hidalgo G, Baldassano RN, Anokhin AP, Heath AC, Warner B, Reeder J, Kuczynski J, Caporaso JG, Lozupone CA, Lauber C, Clemente JC, Knights D, Knight R, Gordon JI (2012) Human gut microbiome viewed across age and geography. Nature 486:222–227. 10.1038/nature11053 22699611 10.1038/nature11053 PMC3376388

[179] Zeng W, Liu P, Pan W, Singh SR, Wei Y (2015) Hypoxia and hypoxia inducible factors in tumor metabolism. Cancer Lett 356:263–267. 10.1016/j.canlet.2014.01.032 24508030 10.1016/j.canlet.2014.01.032

[180] Zhang JC, Chen WD, Alvarez JB, Jia K, Shi L, Wang Q, Zou N, He K, Zhu H (2018) Cancer immune checkpoint blockade therapy and its associated autoimmune cardiotoxicity. Acta Pharmacol Sin 39:1693–1698. 10.1038/s41401-018-0062-2 29991709 10.1038/s41401-018-0062-2 PMC6289335

[181] Zhang X, Gan Y, Zhu H, Liu Z, Yao X, Cheng C, Liu Z, Su C, Zou J (2023) Role of mitochondrial metabolism in immune checkpoint inhibitors-related myocarditis. Front Cardiovasc Med 10:1112222. 10.3389/fcvm.2023.1112222 36760573 10.3389/fcvm.2023.1112222 PMC9902768

[182] Zhao J, Wang H, Huang Y, Zhang H, Wang S, Gaskin F, Yang N, Fu SM (2015) Lupus nephritis: glycogen synthase kinase 3beta promotion of renal damage through activation of the NLRP3 inflammasome in lupus-prone mice. Arthritis Rheumatol 67:1036–1044. 10.1002/art.38993 25512114 10.1002/art.38993 PMC4731039

[183] Zhou W, Mukherjee P, Kiebish MA, Markis WT, Mantis JG, Seyfried TN (2007) The calorically restricted ketogenic diet, an effective alternative therapy for malignant brain cancer. Nutr Metab (Lond) 4:5. 10.1186/1743-7075-4-5 17313687 10.1186/1743-7075-4-5 PMC1819381

[184] Zhou YW, Zhu YJ, Wang MN, Xie Y, Chen CY, Zhang T, Xia F, Ding ZY, Liu JY (2019) Immune checkpoint inhibitor-associated cardiotoxicity: current understanding on its mechanism diagnosis and management. Front Pharmacol 10:1350. 10.3389/fphar.2019.01350 31849640 10.3389/fphar.2019.01350 PMC6897286

[185] Zimmer HG (1992) The oxidative pentose phosphate pathway in the heart: regulation, physiological significance, and clinical implications. Basic Res Cardiol 87:303–316. 10.1007/BF00796517 1384463 10.1007/BF00796517

[186] Zito C, Manganaro R, Ciappina G, Spagnolo CC, Racanelli V, Santarpia M, Silvestris N, Carerj S (2022) Cardiotoxicity induced by immune checkpoint inhibitors: what a cardio-oncology team should know and do. Cancers (Basel). 10.3390/cancers14215403 36358830 10.3390/cancers14215403 PMC9653561

